# PRMT5 inhibition shows in vitro efficacy against H3K27M-altered diffuse midline glioma, but does not extend survival in vivo

**DOI:** 10.1038/s41598-023-48652-x

**Published:** 2024-01-03

**Authors:** Elizabeth J. Brown, Leire Balaguer-Lluna, Adam P. Cribbs, Martin Philpott, Leticia Campo, Molly Browne, Jong Fu Wong, Udo Oppermann, Ángel M. Carcaboso, Alex N. Bullock, Gillian Farnie

**Affiliations:** 1 Nuffield Department of Medicine, Centre for Medicines Discovery, University of Oxford, Oxford, UK; 2 SJD Pediatric Cancer Center Barcelona, Hospital Sant Joan de Deu, Institut de Recerca Sant Joan de Deu, Barcelona, Spain; 3 Nuffield Department of Orthopedics, Rheumatology and Musculoskeletal Sciences, Botnar Research Centre, National Institute of Health Research Oxford Biomedical Research Unit (BRU), University of Oxford, Oxford, UK; 4 Oxford Centre for Translational Myeloma Research, University of Oxford, Oxford, UK; 5 Department of Oncology, Experimental Cancer Medicine Centre, University of Oxford, Oxford, UK; 6 Present Address: Cancer Research Horizons, The Francis Crick Institute, London, UK

**Keywords:** Target identification, Target validation, Drug development, Targeted therapies, CNS cancer, Paediatric cancer

## Abstract

H3K27-altered Diffuse Midline Glioma (DMG) is a universally fatal paediatric brainstem tumour. The prevalent driver mutation H3K27M creates a unique epigenetic landscape that may also establish therapeutic vulnerabilities to epigenetic inhibitors. However, while HDAC, EZH2 and BET inhibitors have proven somewhat effective in pre-clinical models, none have translated into clinical benefit due to either poor blood–brain barrier penetration, lack of efficacy or toxicity. Thus, there remains an urgent need for new DMG treatments. Here, we performed wider screening of an epigenetic inhibitor library and identified inhibitors of protein arginine methyltransferases (PRMTs) among the top hits reducing DMG cell viability. Two of the most effective inhibitors, LLY-283 and GSK591, were targeted against PRMT5 using distinct binding mechanisms and reduced the viability of a subset of DMG cells expressing wild-type *TP53* and mutant *ACVR1*. RNA-sequencing and phenotypic analyses revealed that LLY-283 could reduce the viability, clonogenicity and invasion of DMG cells in vitro, representing three clinically important phenotypes, but failed to prolong survival in an orthotopic xenograft model. Together, these data show the challenges of DMG treatment and highlight PRMT5 inhibitors for consideration in future studies of combination treatments.

## Introduction

Brain tumours are the leading cause of cancer-related deaths in children^1,2^. H3K27-altered Diffuse Midline Glioma (DMG)^3^, formerly known as Diffuse Intrinsic Pontine Glioma (DIPG), is a universally fatal paediatric brainstem tumour which contributes to this figure disproportionately^4^. Over the progression of the disease, cells spread from the pons to infiltrate the midbrain, medulla, cerebellum or thalamus (in over 50% of cases) and the frontal cortex in 25% of cases, with occasional metastasis to the spinal cord^5,6^. The anatomical location precludes surgical resection, while radiotherapy only transiently delays progression and there are currently no effective chemotherapies^7,8^. Thus, the median survival of 9 months has not improved in decades. Given that the median age of diagnosis is 6–7 years, this represents thousands of years of life lost annually^9,10^.

Nearly all H3K27-altered DMG cases carry a p.Lys27Met mutation in either histone H3.3 or H3.1^11,12^. Epigenetic regulation through histone modifying enzymes is important to shape brain development and the ubiquity of a single histone mutation highlights the importance of epigenetics in the development of these tumours. H3K27M mutation leads to global histone H3 hypomethylation and hyperacetylation, contrasted with retention of hypermethylation at tumour suppressor loci that are Polycomb targets^13–15^. Thus, H3K27M mutation sets up a unique epigenetic landscape that may also establish potential therapeutic vulnerabilities. In addition to histone H3, there are also more heterogeneous alterations in the p53 pathway, including mutations in *TP53* and *PPM1D*, as well as in growth factor kinase pathways, including *ACVR1*, *PDFGRA* and *PIK3CA*^16–19^.

A number of histone modifying enzymes have been explored as therapeutic targets in DMG. Restoration of H3K27 methylation by the KDM6 demethylase inhibitor GSK-J4 reduced the viability of H3K27M DMG cells in vitro and extended survival of mice implanted with orthotopic xenografts^20^. Viability loss has also been observed using EZH2 methyltransferase inhibitors to block the hypermethylation of tumour suppressor loci^14^. Hyperacetylated H3 subunits in DMG cells show an enrichment of BET bromodomains BRD2 and BRD4, which promote active transcription^21^. Preventing BRD2/4 recruitment with the small molecule JQ1 reduced tumour size and extended survival in a DMG xenograft model^21^. A screen of 83 drugs of relevance for neuro-oncology also identified DMG cell sensitivity to the HDAC inhibitor panobinostat^22^. Treated cells showed a dose-dependent increase in H3 acetylation, as well as an unexpected increase in H3K27 trimethylation, which resulted in the normalization of the K27M gene expression signature^22^. These effects translated into significantly prolonged survival in mouse models of DMG compared to vehicle controls^22^. In addition, epigenetic drug combinations have demonstrated enhanced cytotoxicity, for example using panobinostat and GSK-J4^22^, EZH2 inhibitor and JQ1^23^, or Corin, a bifunctional inhibitor of HDACs and LSD1^24^.

Protein arginine methyltransferase 5 (PRMT5) catalyzes the transfer of symmetric dimethylation onto a range of proteins, notably histones, which allows dynamic regulation of processes including DNA damage, the cell cycle and neural differentiation^25^. PRMT5 can repress transcription by dimethylating either histone H4 (H4R3me2s) or histone H3 (H3R8me2s)^26,27^. Conversely, cross talk between PRMT5 and other epigenetic pathways can activate gene expression by dimethylating H3R2 leading to recruitment of mixed-lineage leukemia (MLL) and deposition of H3K4me3^28^. Within the brain, PRMT5 regulates neural stem cell survival and oligodendrocyte maturation^29,30^. The overexpression of PRMT5 in adult glioma^31,32^ and the ability of PRMT5 inhibition to deplete cancer stem cells^33^ position PRMT5 as an interesting therapeutic candidate for DMG.

While HDAC, EZH2 and BET inhibitors have proven somewhat effective in numerous pre-clinical models, these treatments have failed to translate into clinical benefit due to either poor blood–brain barrier penetration, lack of efficacy or toxicity^34,35^. Thus, there remains an urgent need for new DMG treatments. Here, we performed a screen of an epigenetic inhibitor library to explore the potential for other drug intervention strategies. From this, we identified inhibition of protein arginine methyltransferase 5 (PRMT5) as an effective treatment to reduce the viability of a subtype of DMG cells in vitro. We further showed that the PRMT5 inhibitor LLY-283 could reduce the stemness and invasive behaviour of DMG cells. Finally, we found that although LLY-283 failed to prolong survival in an orthotopic xenograft model, it did show some reduced infiltration of DMG cells into the mouse forebrain.

## Results

### PRMT5 inhibition potently reduces DMG cell viability

**Figure 1:**
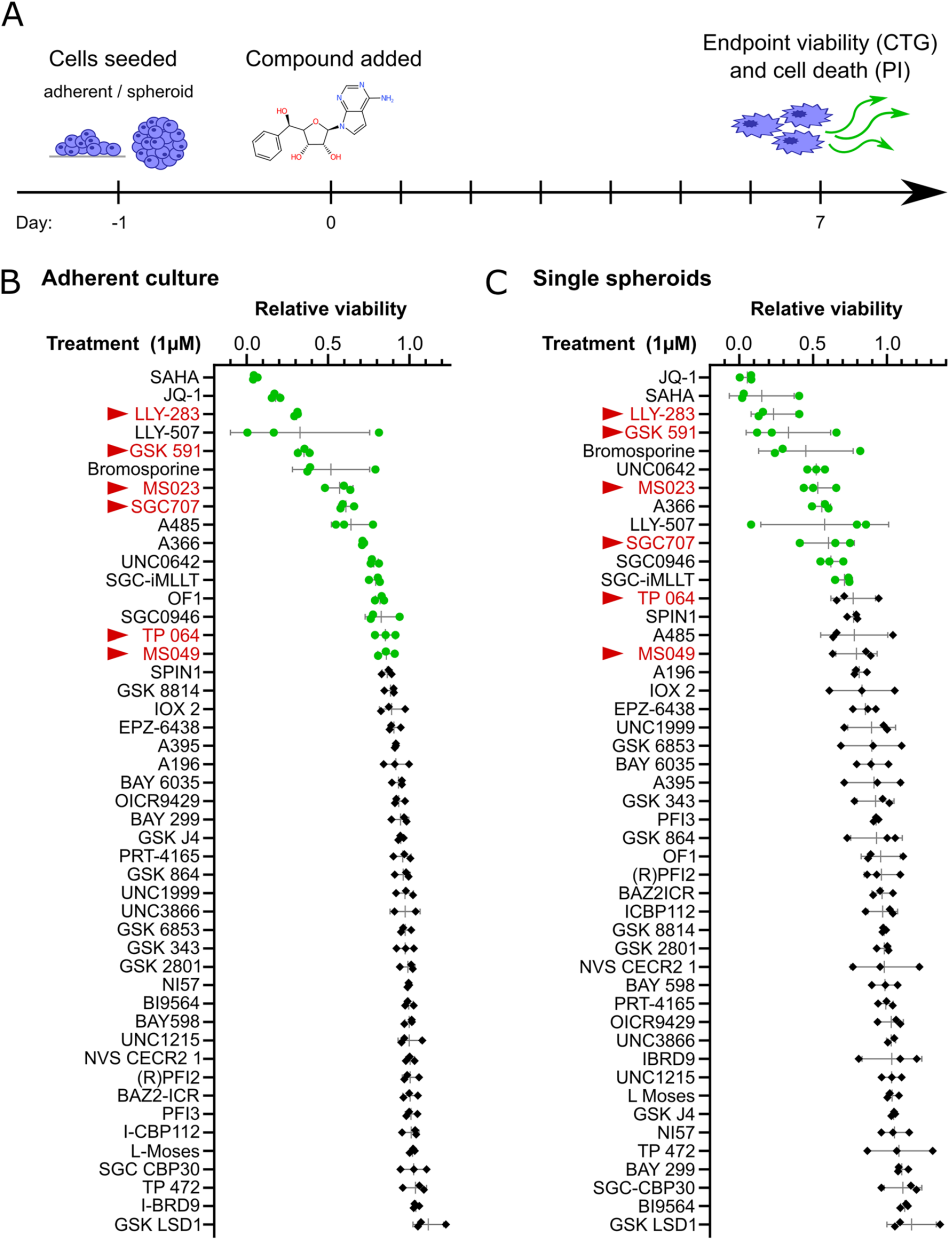
An epigenetic probe screen identified the PRMT family as necessary for DMG viability. (**A**) An overview of the method for viability screening of the epigenetic probe set. HSJD-DIPG-007 cells were seeded into one of two screens (either to grow adherently or as spheroids). The following day each inhibitor was added to a final concentration of 1 µM, alongside a DMSO vehicle control. After 7 days of treatment, the relative viability was measured with the CellTiter-Glo assay. (**B**,**C**) Relative viability of HSJD-DIPG-007 adherent cell cultures after 7 days of growth in the presence of 1 µM of the indicated epigenetic inhibitor. Data points are the mean of 3 (**B**) or 4 (**C**) technical repeats annotated with the SD. Data points for probes that significantly reduced HSJD-DIPG-007 viability are depicted as green circles (multiple two-tailed t-tests, FDR = 1%). The names of PRMT family probes are highlighted in red and marked with a triangle.

To explore potential vulnerabilities in H3K27-altered DMG, we screened a focused set of 47 small molecule epigenetic inhibitors (‘epigenetic probes’) for their ability to reduce cell viability using the patient-derived cell line HSJD-DIPG-007 (Fig. 1A, Supplementary Table 1 and 2). Adherent, as well as spheroid cultures, were treated with 1 µM of each epigenetic probe for 7 days, corresponding to the recommended dose and assay length for the probe set. This allows time for epigenetic mark modulation, as well as the subsequent phenotypic changes^36,37^, while the use of both 2D and 3D culture methods may be more predictive of physiological behaviour^38^. Of the probes tested, 16 and 12 markedly reduced the viability of adherent and spheroid DMG cultures, respectively (Fig. 1B, C). These included the BRD4 inhibitor JQ-1 and HDAC inhibitor SAHA, both previously characterized in DMG models^21,39–41^, as well as inhibitors of p300 and the PRMT family methyltransferases. Of note, all 6 of the PRMT probes tested reduced cell viability and 4 reduced the viability of both adherent and spheroid cultures > 50%. The two most effective PRMT-specific probes, LLY-283 and GSK591, were targeted against PRMT5 and by distinct molecular mechanisms, adding confidence to the hit identification. GSK591 is a peptide substrate competitive inhibitor, whereas LLY-283 binds to the SAM-binding pocket^37,42^. From these observations, PRMT inhibition was selected as the ongoing focus of study.

**Figure 2:**
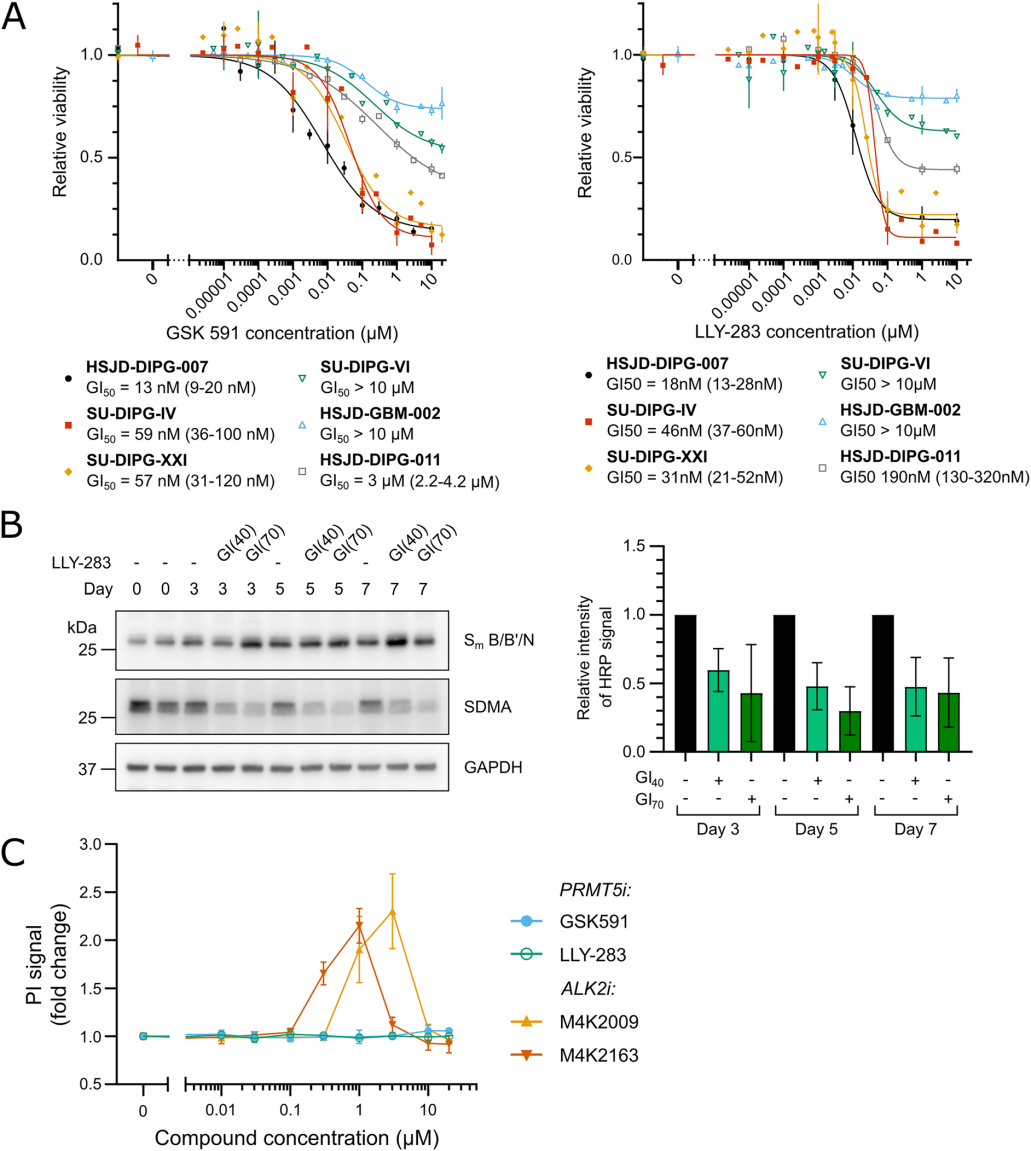
PRMT5 inhibition potently reduces the viability of multiple DMG cell lines. (**A**) The relative viability of DMG single spheroids formed from the indicated DMG cell line* after a 7 day treatment with the PRMT5 inhibitors GSK591 (left) or LLY-283 (right). GI_50_ values were interpolated from each growth inhibition curve. (* except for SU-DIPG-IV which grows as a monolayer). (**B**) Left, the dimethylation of S_m_ B/B’/N after treatment with the 40% growth inhibitory concentration (GI_40_) of LLY-283 visualised by immunoblot (representative of 3 independent repeats). The cropped images span the entire width of the membrane and retain all samples included on the gel. To allow visualisation of multiple post translational modifications and proteins of different molecular weights, membranes were stripped and re-stained with different antibodies as described in the methods. Uncropped Western blots are shown in Supplementary Fig. 2. Right, quantification of band intensity of all 3 independent repeats. (**C**) SU-DIPG-IV cells were treated with the indicated concentration of the PRMT5 inhibitors GSK591 or LLY-283, or ACVR1 inhibitors M4K2009 or M4K2163 for 7 days in the presence of 1 µg/mL PI. Fluorescence signal was normalised to the vehicle control for each compound. Data points represent the mean of 3 independent repeats ± SD.

For validation, the effect of the PRMT5 inhibitors LLY-283 and GSK591 on cell viability was confirmed using full growth inhibition curves in five patient-derived H3K27M DMG cell lines, as well as in a sixth paediatric glioma line with a H3G34R mutation (Fig. 2A). Normal cell lines were not included as previous studies have shown that normal human astrocytes are insensitive to PRMT5 inhibition, whereas foetal neural stem cells have GI_50_ values of 7–155 nM, comparable to those of adult glioma^32^. Other PRMT family inhibitors were tested similarly in 2 DMG cell lines (Supplementary Fig. 1). Most notably, PRMT5 inhibitors LLY-283 and GSK591 displayed low nanomolar GI_50_ values in all three H3K27M DMG cell lines with mutant *ACVR1*/wild-type *TP53* status, the lowest being 13 nM for GSK591 in HSJD-DIPG-007 (Fig. 2A). Loss of PRMT5 function was confirmed by a reduction in symmetric dimethyl arginine (SDMA) on S_m_ B/B’/N, a well characterized target of PRMT5^43^ (Fig. 2B and Supplementary Fig. 2). Mutant *ACVR1*/wild-type *TP53* cell lines were sensitive to PRMT5 inhibition irrespective of whether they displayed H3.3 or H3.1 mutation (Supplementary Table 3) suggesting that the phenotype was independent of the H3 isoform. By contrast, H3K27M and H3G34R lines with wild-type *ACVR1/*mutant *TP53* status displayed GI_50_ values > 10 µM for all PRMT inhibitors tested (Fig. 2A and Supplementary Fig. 2). An intermediate response was found for the H3K27M line HSJD-DIPG-11 with wild-type *ACVR1* and wild-type *TP53* (LLY-283 GI_50_ = 190 nM; GSK591 GI_50_ = 3 µM). Together these data show that DMG lines with wild-type *TP53* were generally > 100-fold more sensitive to PRMT5 inhibition than those with mutant *TP53*, particularly in a background of mutant *ACVR1*.

The mode by which LLY-283 and GSK591 reduced DMG cell viability was further characterized using propidium iodide (PI) staining for cell death. Comparison of PRMT5 inhibition and ACVR1 kinase inhibition, a positive control for apoptotic cell death^44^, revealed different effects on viability (Fig. 2C). Treatment with the recently described ACVR1 kinase inhibitors M4K2009 and M4K2163^45,46^ resulted in marked PI staining, as expected for cell death (Fig. 2C). However, no change in PI staining was observed upon treatment with LLY-283 or GSK591, suggesting instead a growth arrest (Fig. 2C).

Overall, these data show that PRMT5 inhibition can potently reduce the viability of multiple patient-derived DMG cell lines carrying a range of disease-causing mutations and wild-type *TP53*. LLY-283 was selected for continued investigation based on its known blood–brain barrier penetrance and previous efficacy in an orthotopic mouse model of glioblastoma^32^.

### PRMT5 inhibition alters transcription of differentiation and extracellular matrix associated genes in vitro

**Figure 3:**
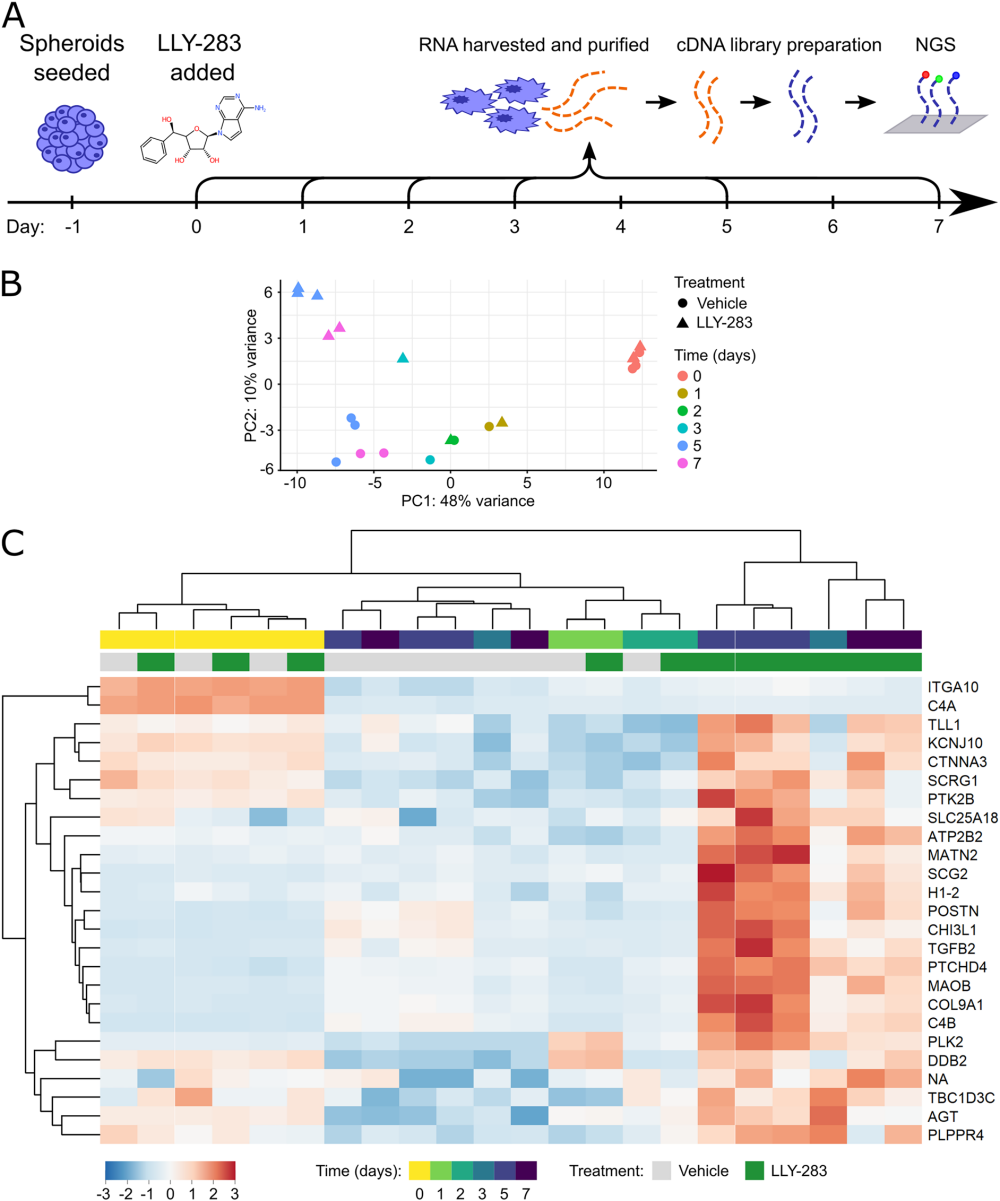
PRMT5 inhibition altered gene expression after 3 days of treatment. (**A**) HSJD-DIPG-007 cells were seeded as spheroids and treated with the LLY-283 GI_40_ the following day. Total cellular RNA was collected after a 0, 1, 2, 3, 5, 7 or 10 days treatment with LLY-283. RNA was collected and sequenced for 3 technical repeats for days 0 and 5, 2 technical repeats for day 7 and 1 technical repeat for days 1, 2 and 3. (**B**) The clustering of samples according to the 1st and 2nd principal components. Gene expression changes over time and diverges after 3 days along PC2 according to treatment group. (**C**) The relative expression of the 25 genes with the highest PC2 weighting (scaled by row). Column clustering shows the similarity between day 0 and vehicle samples, and the differential expression within later treated samples.

**Figure 4:**
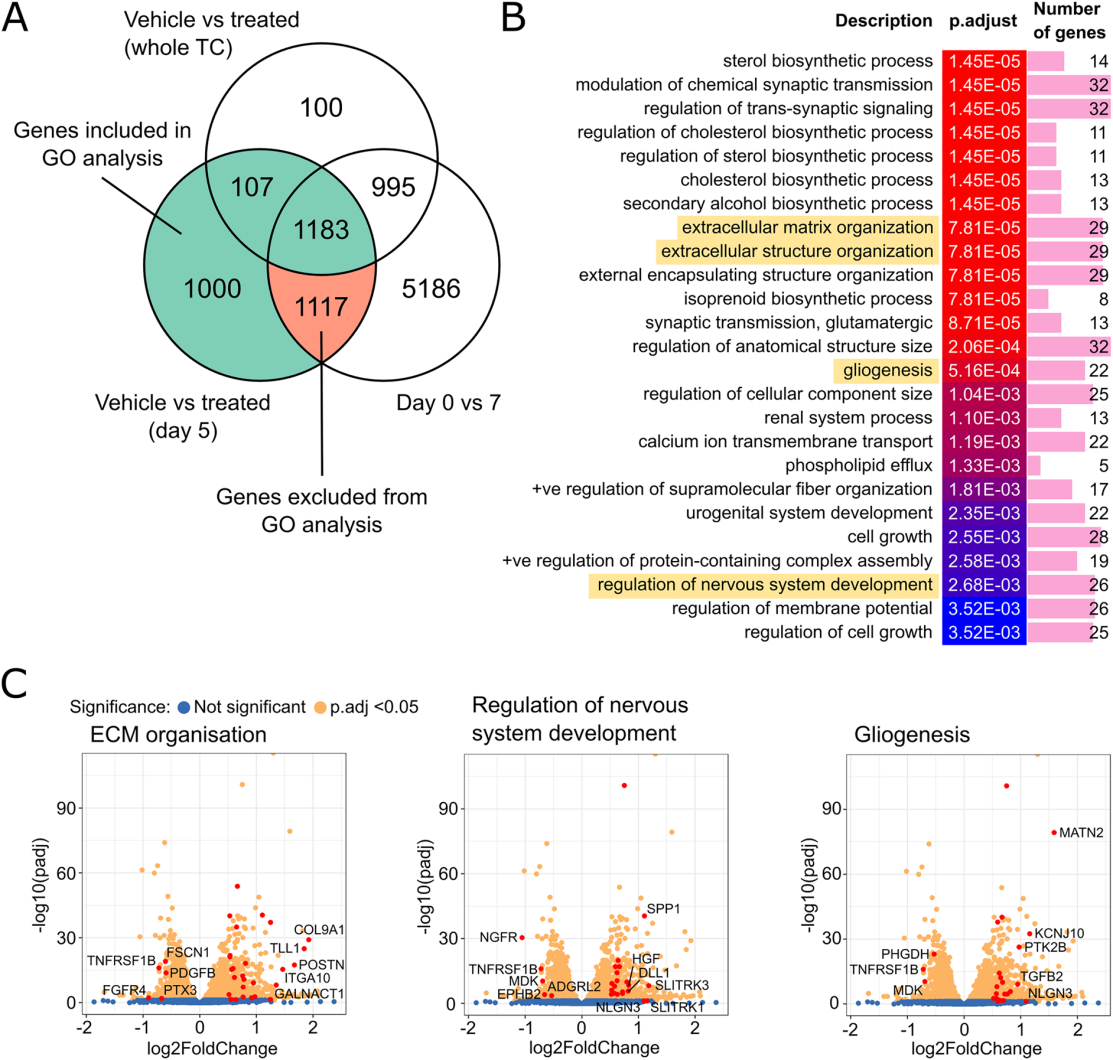
PRMT5 inhibition alters ECM organisation and differentiation of DMG cells. (**A**) The overlap between the genes differentially expressed between treatment groups for day 5 samples only, between treatment groups over the whole time course, or between day 0 and day 7. (**B**) The top 25 significant gene ontology (GO) terms enriched in the list of genes that were differentially expressed between treatment groups at day 5 (excluding those that were differentially expressed between days 0 and 7, but not the full time course. Similar terms have been removed). Terms selected for further validation are highlighted in yellow. (**C**) Volcano plots of differentially expressed genes at day 5 of the LLY-283 treatment where significantly changed genes are coloured yellow (padj < 0.05). Genes associated with the indicated GO terms are highlighted in red.

PRMT5 regulates a wide range of epigenetic and intracellular signalling pathways. We performed bulk RNA-sequencing to simultaneously assess which of these processes were altered within DMG cells after PRMT5 inhibition. The PRMT5 inhibition sensitive cell line, HSJD-DIPG-007, was treated with a single dose of LLY-283 (GI_40_) and the transcriptome assessed at 5 time points (Fig. 3A). This cell line and compound have the advantage that they are both compatible with in vivo experiments (discussed below), while the GI_40_ dosing was used to assess transcriptomic changes in the absence of high levels of cell death. Principal component (PC) analysis of the transcriptome of each sample showed the divergence of gene expression between vehicle and treated groups after 3 days + /− LLY-283 treatment (Fig. 3B, C). Unsupervised hierarchical clustering of the samples according to the top weighted genes within PC2 confirms that samples only cluster according to treatment after day 3 (Fig. 3C). PC1 accounted for 48% of the variance between samples and differentiated primarily according to time, whereas PC2 accounted for 10% of variance and separated samples according to treatment group (Fig. 3B). Despite the large variance between samples at different time points, from day 3 onwards samples cluster primarily according to treatment group suggesting that, when comparing treatment groups, time was a minimally confounding factor. The greatest variance between transcriptomes according to treatment group was observed at days 5 and 7 and so differential expression analysis was performed on day 5 samples. DESeq2-based analysis found 3085 genes that were differentially expressed (DE) between vehicle and treated samples (false discovery rate (FDR) < 0.01). Analysis of the full time course with DESeq2 found 1566 differentially expressed genes (FDR < 0.01), whereas a comparison of day 0 to day 7 gene expression found 5790 DE genes (FDR < 0.01). 60% of the genes that were differentially expressed at day 5 were downregulated, in contrast to previous observations that PRMT5 mediated methylation of histones mainly represses transcription^26,27^. *PRMT5* had a mean of 1580 reads for all samples and was not differentially expressed between treatment groups throughout the time course (padj = 0.14). Gene ontology (GO) analysis was performed on genes differentially expressed at day 5 between treatment groups, excluding those which were differentially expressed between days 0 and 7 but not the full time course (Fig. 4A). Genes differentially expressed at day 5 between treatment groups were enriched with transcripts associated with metabolism, differentiation and extracellular matrix (ECM) organisation, (Fig. 4B,C ), with similar terms enriched in the time course result set (Supplementary Fig. 3). KEGG enrichment analysis also highlighted changes in cellular metabolism (steroid and terpenoid biosynthesis, padj < 0.05, Supplementary Table 4). The role of PRMT5 in regulating metabolism and differentiation is well established^25,47^ so the differential expression of genes associated with these terms was selected for validation by RT-qPCR. This confirmed the differential expression of 7/10 of the highest fold change transcripts from the ‘Cholesterol metabolism’**,** ‘Negative regulation of neural differentiation’ and ‘Gliogenesis’ terms (Supplementary Fig. 4).

**Figure 5:**
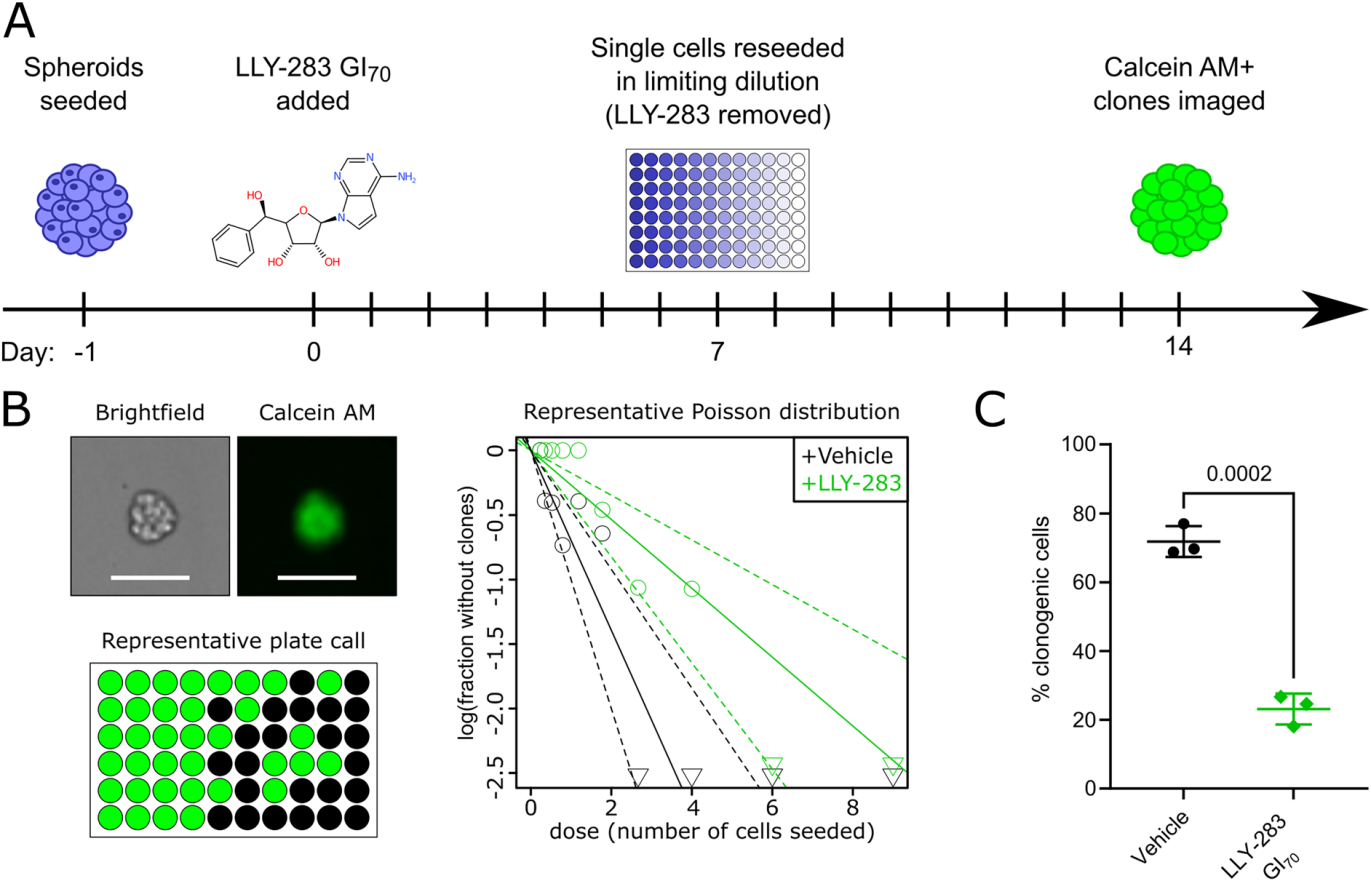
PRMT5 inhibition impairs clonogenicity of DMG cells. (**A**) HSJD-DIPG-007 cells were seeded as loose spheroids. The following day the LLY-283 GI_70_ was added to the growth medium. After a 7-day treatment with LLY-283 GI_70_ spheroids were split and re-seeded as single cells in a titration (6 technical repeats per seeding density) which allows derivation of the fraction of clonogenic cells in the population. After 7 days calcein AM was added to the growth medium and viable clones were imaged. (**B**) Left to right: Representative brightfield and fluorescent images of a HSJD-DIPG-007 clone, an overview of calls (green where at least one clone is present) of one 96 well plate and a Poisson distribution calculated from one independent limiting dilution assay. (**C**) The percentage of clonogenic cells, + /− a 7-day pre-treatment with LLY-283 GI_70_. Data points are the estimated stem cell fraction from each independent repeat, annotated with mean and SD. Statistical significance (*p* = 0.0002) was calculated by unpaired t-test.

The role of PRMT5 in differentiation and stemness of neural cells is well characterised^25^. In accordance with this, genes that were upregulated following PRMT5 inhibition, such as Matrilin-2 (*MATN2*), apolipoprotein E (*APOE*) and Potassium Inwardly Rectifying Channel Subfamily J Member 10 (*KCJN10*) were associated with differentiation of neural lineages^48–50^. Whereas genes downregulated following PRMT5 inhibition such as midkine (*MDK*), nerve growth factor receptor (*NGFR*) or ephrin type-B receptor 2 (*EPHB2*) were associated with stemness^51–53^. To link these transcriptomic changes to a functional phenotype we tested for subsequent changes in the stem-like behaviour of DMG cells using an extreme limiting dilution assay (ELDA) in vitro^54,55^. Following a 7-day pre-treatment with LLY-283 GI_70,_ there was a decrease in the clonogenicity of HSJD-DIPG-007 cells from 72 to 23% of cells able to generate a clone after 7 days (Fig. 5). Thus, in addition to reducing DMG cell growth, PRMT5 inhibition reduced DMG stem-like activity post treatment, which could be a beneficial effect to help prevent tumour re-initiation post treatment.

**Figure 6:**
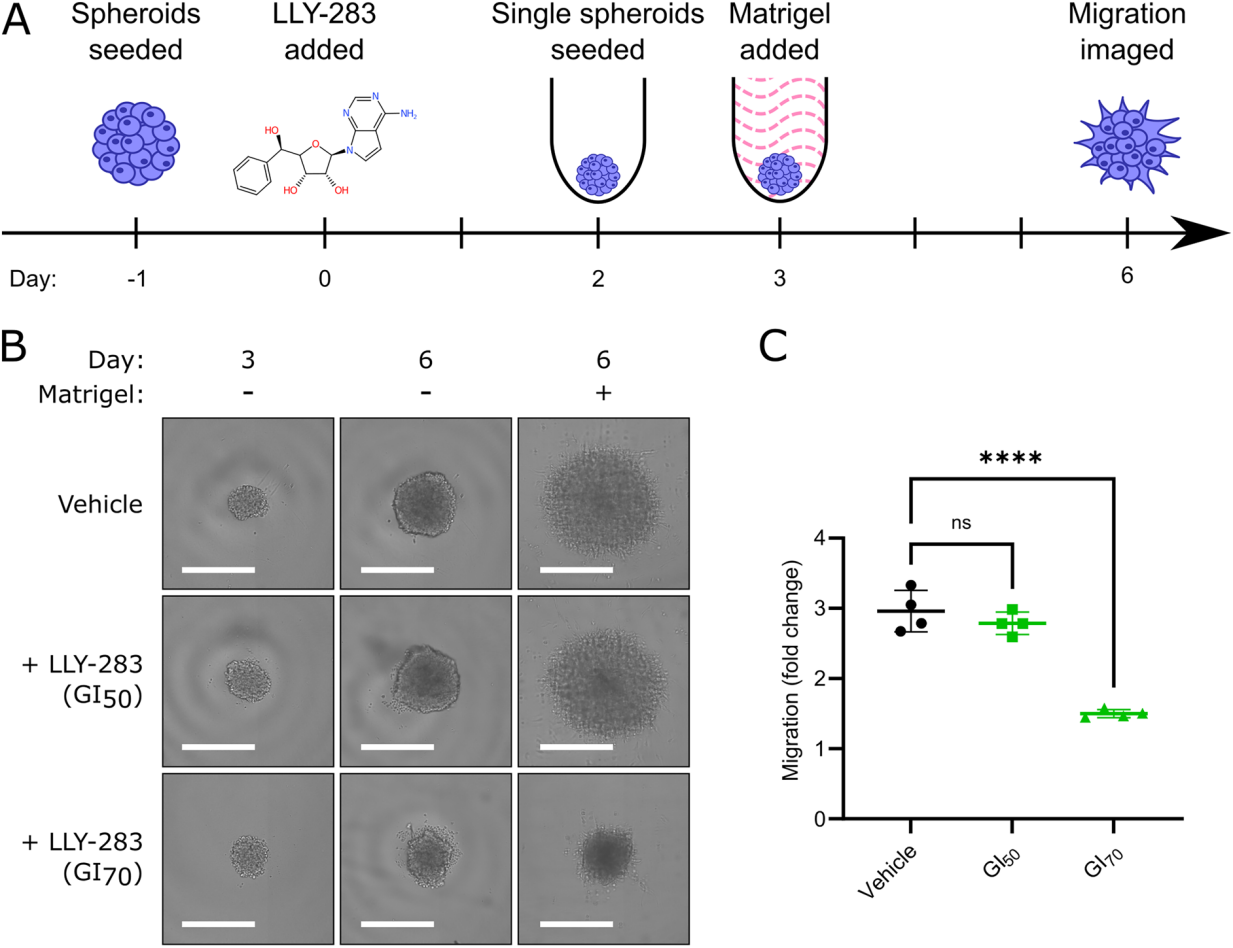
PRMT5 inhibition reduced invasion of DMG cells into Matrigel (**A**) HSJD-DIPG-007 cells were seeded as loose spheroids and the following day were treated with a vehicle control, the LLY-283 GI_40_ or GI_70_. After 2 days HSDJ-DIPG-007 cells were dissociated, counted and re-seeded into ultra-low-attachment U-bottomed 384 well plates (generating single spheroids) in medium containing the same concentration of vehicle/LLY-283. The following day half of the spheroids were embedded in 4.65 mg/mL Matrigel (again maintaining vehicle/LLY-283) and the other half remained as controls for undisturbed spheroid growth. Invasion into Matrigel was imaged by brightfield after a further 3 days for a total treatment period of 6 days. (**B**) Representative images of spheroids before Matrigel addition (day 3) and at 3 days + /− Matrigel addition (day 6) showing characteristic outward invasion of HSJD-DIPG-007 cells. Scale bars represent 500 µm. (**C**) Quantification of HSJD-DIPG-007 invasion into the surrounding Matrigel expressed as the fold change in overhead cross-sectional area compared to the without Matrigel spheroid growth control. ****p < 0.0001 by ordinary one-way ANOVA with Dunnett’s multiple comparisons test.

PRMT5 has been linked previously to the regulation of focal adhesion and the cytoskeleton^56^ and promotes invasive growth of colorectal cancer^57^. Many of the suppressed genes in the ‘ECM organisation’ GO term have known roles in cell invasion; for example, FGFR4 (Fibroblast Growth Factor Receptor 4), Fascin-1 (FSCN-1), Platelet Derived Growth Factor Beta (PDGF-B) and Pentraxin 3 (PTX3) mediate invasion in multiple solid tumours, including glioma^58–61^. To test whether a reduction of transcripts associated with tumour cell invasion translates into a change in the migratory phenotype of DMG cells, we treated HSJD-DIPG-007 tumourspheres in vitro with LLY-283 and measured their invasion into Matrigel (Fig. 6). The embedding of spheroids into Matrigel triggered invasive growth and a threefold greater increase in cross-sectional area relative to growth in the absence of extracellular matrix (Fig. 6C). By contrast, spheroids treated with LLY-283 GI_70_ increased in area by only 1.5-fold (*p* < 0.0001, Fig. 6C). Together the data presented show that PRMT5 inhibition can reduce viability, stem-like activity and invasion of DMG cells in vitro, representing three clinically important phenotypes.

### PRMT5 inhibition with LLY-283 does not prolong survival in vivo

**Figure 7:**
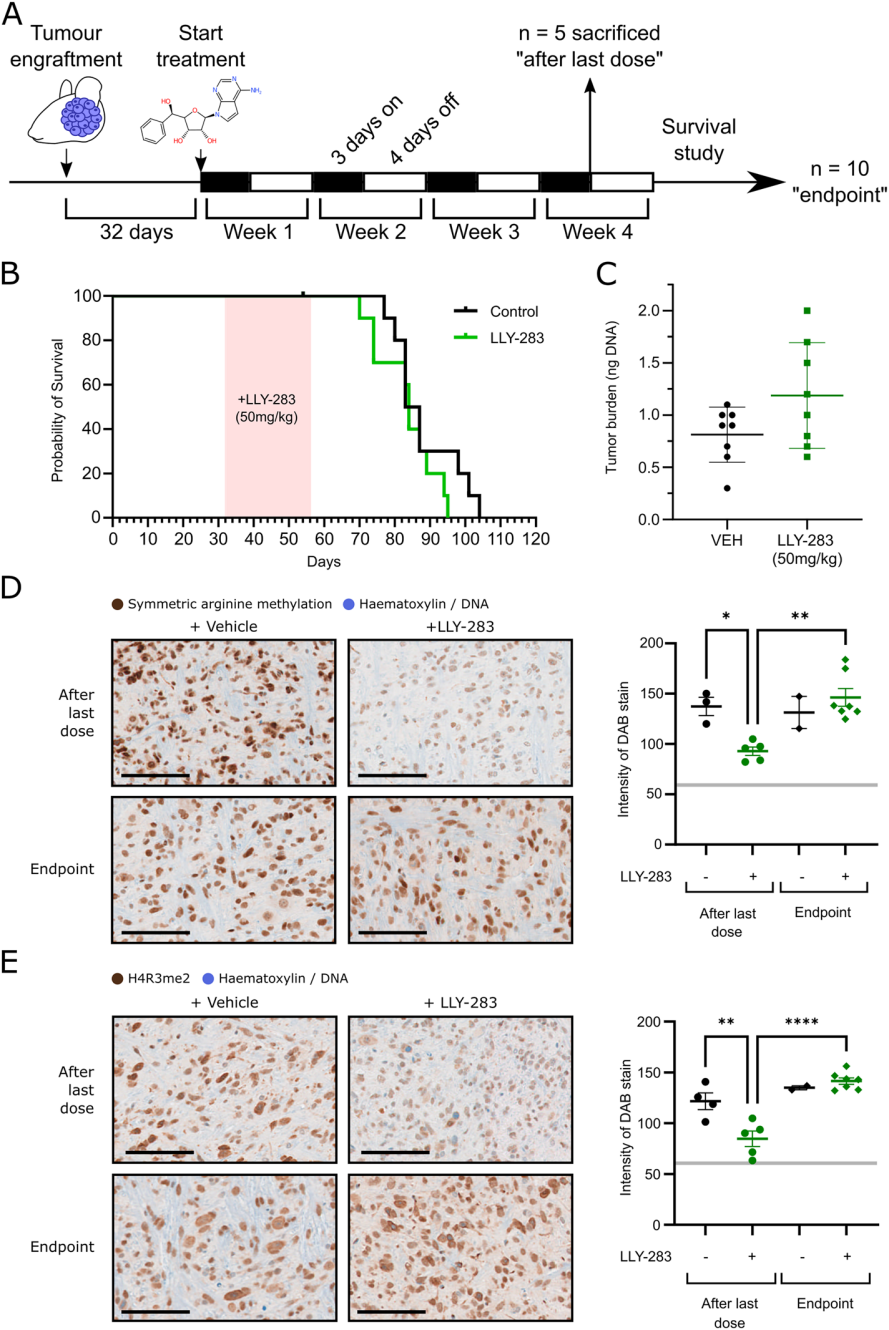
PRMT5 inhibition did not improve survival in a patient dervied xenograft model of DMG (**A**) Athymic nude mice were engrafted with HSJD-DIPG-007 cells. Tumours were allowed to initiate for 32 days before vehicle (n = 15) or LLY-283 (n = 15) was administered at 50 mg/kg by oral gavage, 3 days on, 4 days off for 4 weeks. After 4 weeks 5 mice from each group were sacrificed, and the remaining were used to measure survival (endpoint criteria: 20% weight loss due to disease). (**B**) Survival of mice after 4 week treatment with vehicle or LLY-283. The treatment period is highlighted in red. Median survival for each group was compared by log-rank (Mantel-Cox test, *p* = 0.2) (**C**) Tumour burden as indicated by the amount of R206H *ACVR1* DNA present in the brains of mice sacrificed immediately after administration of the final LLY-283 dose. (**D**) Left: Representative images of mouse brainstems harvested after the last dose of LLY-283 or at the endpoint, + /− 50 mg/kg LLY-283 that have been stained for symmetric arginine dimethylation (**D**) or H4R3me2 (**E**) (counterstained with haematoxylin). Scale bars represent 100 µm. Right: Quantification of the intensity of DAB staining in the nuclei within the brainstem. Dashed line indicates the intensity of staining in the IgG isotype control. Significance was determined by one way ANOVA with Tukey’s multiple comparison test (* p < 0.05; ** p < 0.01; **** p < 0.0001).

The dire prognosis for DMG patients warrants rapid testing of novel therapeutic targets in in vivo models. LLY-283 was selected over GSK591 for in vivo testing as it has been previously demonstrated to penetrate CNS tissues at a concentration of 119 nM, 24 h after a 50 mg/kg dose^32^. To our knowledge, GSK591 has not been characterized for use against CNS malignancies in vivo. PRMT5 inhibition using LLY-283 was therefore tested for its ability to extend survival in an established xenograft model of DMG^44^. According to a previously described dosing strategy^32^, established HSJD-DIPG-007 brainstem xenografts (n = 15 per group) were treated with 50 mg/kg LLY-283 or vehicle by oral gavage, 3-days-on 4-days-off, for 4 weeks (Fig. 7A). This dosing strategy was previously developed to avoid the > 20% weight loss induced by LLY-283 treatment on alternate days^32^. 5 mice from each treatment group were sacrificed immediately after the completion of the treatment, and the remaining 10 were used to measure survival (Fig. 7A). Treatment neither prolonged survival nor reduced tumour burden (Fig. 7B, C) and was associated with an additional 4% weight loss over the treatment period (Supplementary Fig. 5, *p* = 0.01, two-tailed t-test). Penetration of LLY-283 into the brainstem was confirmed by testing target engagement two hours post treatment using immunohistochemistry to measure the reduction in two methylation marks known to be mediated by PRMT5 (histone H4R3me2s, and total symmetrical dimethyl arginine (SDMA)) (Fig. 7, Supplementary Fig. 6). Both were partially reduced immediately after end of treatment, however, methylation was restored by the time mice were sacrificed at the end of the study (Fig. 7D,E).

**Figure 8:**
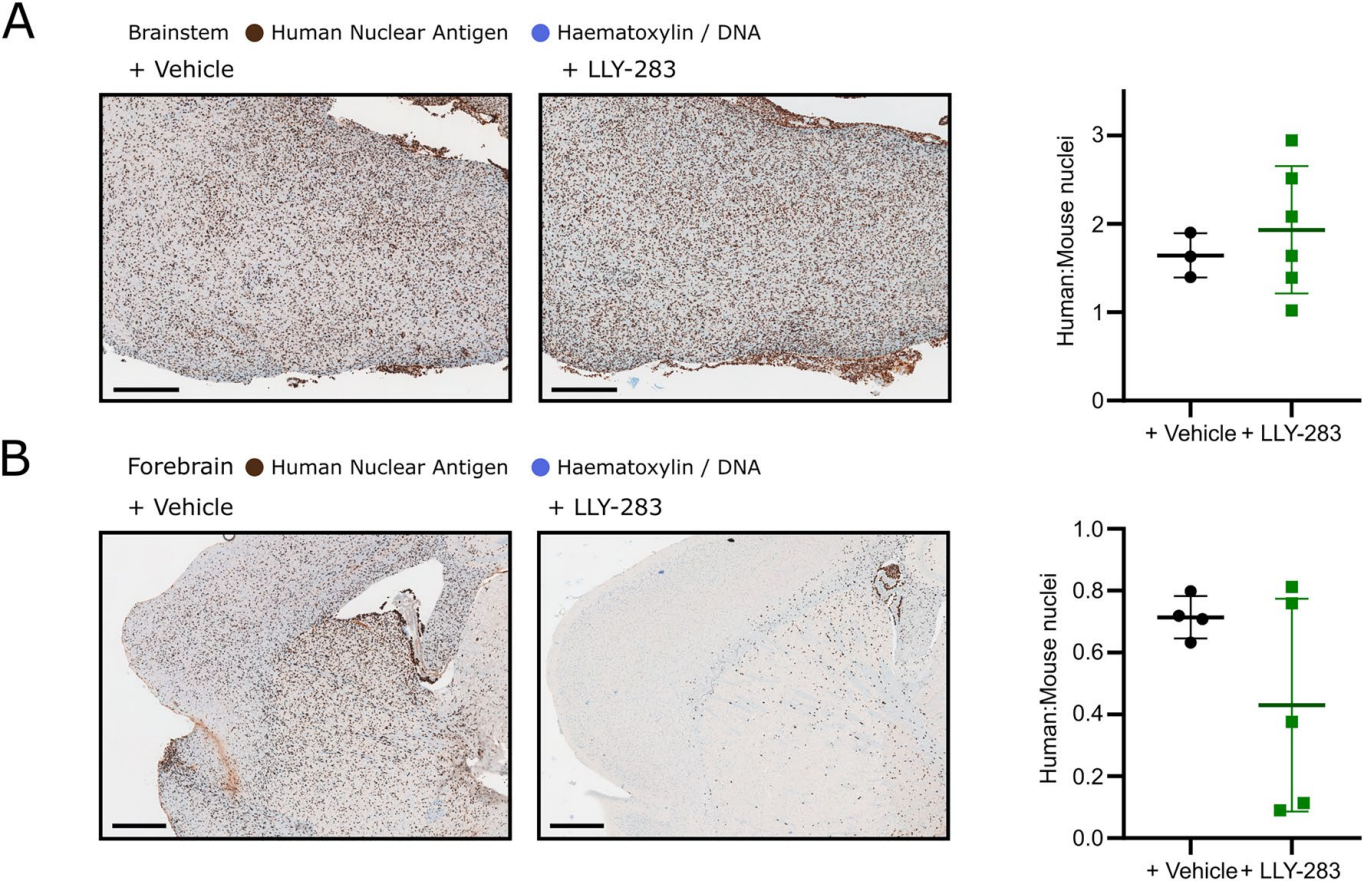
PRMT5 inhibition impaired migration of DMG cells into the mouse forebrain. Left: Representative images of sectioned mouse brainstems (**A**) or forebrains (**B**) harvested immediately after the final dose of vehicle/LLY-283 (50 mg/kg) immunohistochemically stained for human nuclear antigen (counterstained with haematoxylin). Scale bars represent 500 µm. Right: Quantification of the ratio of human:mouse cells in the brainstem (**A**) or forebrain (**B**). Each data point represents one field of view from each biological repeat and each treatment group is annotated with the mean and SD.

Given the reduced invasion seen in vitro with LLY-283, the location of DMG cells in the brains of mice treated with LLY-283 was also examined by staining of tissue sections for human nuclear antigen (Fig. 8). In accordance with ddPCR measurement of tumour burden, the number of human cells in the brainstem did not change after LLY-283 treatment (Fig. 8A). However, the number of cells that invaded into the forebrain was reduced in 3/5 LLY-283 treated mice (Fig. 8B).

Although the potent ability of PRMT5 inhibition to reduce viability in vitro did not translate into improved survival in vivo, the reduction in stem-like activity and migration of DMG cells could still potentially provide benefit for future study of combination treatments.

## Discussion

From a screen of epigenetic chemical probes, we identified PRMT5 inhibition as an effective treatment to reduce the viability of H3K27-altered DMG cell lines in vitro. This finding was supported by the comparable potency of two independent PRMT5 inhibitors acting via distinct molecular mechanisms, as well as the lack of effect of matching negative control compounds (close chemical analogs of LLY-283 and GSK591 inactive against PRMT5). Notably, PRMT5 inhibition reduced viability more potently than EZH2 inhibition (probes EPZ-6438 and GSK343), an epigenetic target previously characterized in DMG models^14,23^. The low nanomolar GI_50_ values of LLY-283 and GSK-591 were observed in H3K27M DMG cell lines with mutant *ACVR1* and wild-type *TP53* status. Estimates suggest this constitutes up to 50% of DMG cases and 87% of H3.1K27M DMG tumours^18,62,63^.

The increased sensitivity of mutant *ACVR1* and wild-type *TP53* DMG cells to PRMT5 inhibition also implies an interaction between the role of PRMT5, ACVR1 and TP53 in DMG cells. PRMT5 has been shown to regulate the p53 response via methylation of p53, altering p53 promoter binding specificity such that cell cycle arrest is initiated over apoptosis^64,65^. Similarly, PRMT-family proteins are recognized cofactors in the BMP/TGF-β signaling pathways associated with ACVR1 and methylate inhibitory SMAD6 and SMAD7 to relieve their inhibition^66,67^. Tests using 240 cancer cell lines have identified *TP53* mutation as the most highly correlated biomarker of resistance to PRMT5 inhibition^68^. However, such correlation was not observed in a focused study of 46 patient-derived glioblastoma lines, which also showed a spectrum of drug sensitivities^32^ and our analysis of the DMG transcriptome did not identify changes in the p53 pathway. Similarly, *MTAP* deletion can sensitise cancer cell lines to PRMT5 inhibition, but there was no correlation between *MTAP* copy number and PRMT5 dependency in adult glioma^32^, and DMG cell lines without *MTAP* deletions exhibited diverse sensitivities to PRMT5 inhibition.

The transcriptomic changes observed upon PRMT5 inhibition in DMG cells suggested multiple mechanisms by which viability could be altered in this system. PRMT5 is known to regulate neural stem cell survival^69^, as well as glial cell differentiation^30^. In the present study, PRMT5 inhibition led to transcriptomic changes associated with reduced stemness and a functional decrease in clone forming potential in vitro. We also observed repression of genes associated with cell invasion, impaired invasion of DMG cells into Matrigel and some indication of reduced invasion in vivo using an orthotopic xenograft model of DMG. PRMT5 activity regulates assembly of the cytoskeleton and focal adhesion^56^. Loss of expression of FGFR4 and integrins, both downregulated after PRMT5 inhibition in DMG, was associated with impaired invasion in colorectal and lung cancer^57,58^. Expression of both genes has previously been shown to be dependent on PRMT5 expression^57,70^. Use of PRMT5 inhibition to reduce invasion of DMG cells into surrounding anatomical structures could limit symptoms associated with progression of DMG or improve efficacy of focal radiotherapy^6^.

The specific mechanism by which PRMT5 inhibition leads to the impaired stemness and invasion of DMG cells remains unclear. However, the large number of differentially expressed genes could suggest that PRMT5 interacts with another epigenetic pathway to alter a range of tumour relevant pathways. Despite the common perception of PRMT5 activity as mediating transcriptional repression via deposition of H4R3me2s or H3R8me2s^26,27^, we observed that the majority of DE genes being repressed after inhibition of PRMT5 activity. This could imply that the crosstalk between PRMT5 and complexes such as MLL or PRC2 which indirectly promote gene expression^28,71,72^, are more relevant within DMG cells.

Unfortunately, systemic administration of LLY-283 did not extend survival in this model, in contrast to a mouse model of adult glioblastoma where LLY-283 was effective^32^. Sachamitr et al*.* demonstrated that LLY-283 remains in the brain at therapeutic concentrations 24 h after a single dose leading to near complete loss of SDMA during treatment. Conversely, this model only showed partial loss of SDMA, possibly reflecting limited penetration to the brainstem specifically relative to the rest of the brain^73,74^. Moreover, arginine methylation was restored by the end point, indicating that treatment may need to be continued beyond four weeks to the end point, as done by Sachamitr et al*.*. As LLY-283 was developed as an experimental probe, use of more recently developed and more ‘drug-like’ molecules, such as TNG908^75^, may enhance brainstem penetration and extend treatment benefits.

H3K27-altered DMG tumours are highly aggressive malignancies diagnosed at grade IV, at which point they progress rapidly to life end^9,76^. As such, these tumours represent a highly challenging cancer type for monotherapies and combination or more complex treatments are likely to be necessary^77–81^. Indeed, the top hit in our initial screen, the HDAC inhibitor SAHA, failed to improve patient outcomes in phase I/II clinical trials^82^.

Inhibitors of PRMTs have been discussed as silver bullets for brain tumour therapy^83^. PRMT5, in particular, has shown promise as a therapeutic target in models of adult glioblastoma^32,84^, neuroblastoma^32,85^ and medulloblastoma^86^. Furthermore, PRMT5 overexpression correlates with histological grade in multiple tumour types, including glioma^31,32,87,88^. Thus, a range of PRMT5 small molecule inhibitors have entered phase I or phase II clinical trials (ClinicalTrials.gov identifiers NCT03886831, NCT04676516, NCT03573310, NCT03854227 and NCT04089449). While not developed for DMG tumours, these studies have potential to yield future brain-penetrant drugs and dosing regimens superior to the pre-clinical compound LLY-283 that could be re-tested in DMG, perhaps with improved target engagement and tolerability for prolonged treatment.

Clinical trials for H3K27-altered DMG typically involve relatively small patient numbers and patient stratification has been rare^89^. However, stratification is likely to increase in future with the development of safe stereotactic biopsies, genomic markers and better targeted treatments^62^. The ability to target stemness is particularly important as aggressive cancer stem cells are likely linked to treatment resistance^90,91^. Inhibition of invasion of DMG cells is similarly important to reduce extrapontine spread and to limit symptoms associated with the infiltration of other anatomical sites^5,6^. Here, the reduced viability, stemness and invasion of mutant *ACVR1*/wild-type *TP53* DMG lines in vitro suggests promise for PRMT5 inhibition in future drug combinations for this most challenging paediatric tumour.

## Methods

### Cell culture

The patient tumour-derived DMG cell lines HSJD-GBM-002, HSJD-DIPG-007, HSJD-DIPG-011, SU-DIPG-IV, SU-DIPG-VI, and SU-DIPG-XXI (listed in Supplementary Table 3) were obtained from Chris Jones and Diana Carvalho (ICR, London)^44^. These cell lines were established from biopsy or autopsy material obtained from DMG and glioma cases from multiple centres^19,34,92^. DMG cell lines were maintained at 37 °C, 5% CO_2_, and were regularly confirmed to be mycoplasma negative by PCR. They were cultured as described in^14,92^. Briefly, cells were cultured in 1:1 neurobasal-A medium, DMEM-F12 (both Gibco) supplemented with HEPES, sodium pyruvate, MEM non-essential amino acids (all Gibco), B-27 (Thermo Fisher), heparin (Sigma), Epidermal Growth Factor (EGF), Fibroblast Growth Factor basic (FGF), platelet derived growth factor (PDGF)-AA, and PDGF-BB (all Peprotech). The epigenetic probe library developed by the Structural Genomics Consortium was purchased from Tocris (see Supplementary Table 1 and 2 for details).

### Viability screen and growth inhibition curves

1500 (adherent screen) or 40 (spheroid screen) HSJD-DIPG-007 cells were seeded per well, and compounds added as a single dose to a final concentration of 1 μM (0.01% DMSO vehicle control) the following day. The CellTiter-Glo (CTG) assay was performed according to manufacturer’s instructions. Briefly, after a 30 min incubation at room temperature, culture lysate was transferred to a white 384-well plate and luminescence was recorded with the ClarioSTAR plate reader. Data were plotted in GraphPad Prism (v8.4.2) and significance determined by multiple t-tests, using a two-stage linear step-up procedure, with Q = 1%^93^.

For growth inhibition curves, 2000 HSJD-GBM-002, SU-DIPG-VI, HSJD-DIPG-011 or SU-DIPG-XXI cells, 175 SU-DIPG-IV or 40 HSJD-DIPG-007 cells were seeded per well (four technical repeats, in 50% final medium volume) to account for different growth rates. The following day, compounds were diluted in culture medium and added as a single dose 1:1 with the existing medium to the concentrations indicated. Vehicle controls were 0.1% (v/v) DMSO. After 7 days of culture the CTG assay was performed as previously described. For each spheroid, viability is expressed relative to the viability of the vehicle control. GI_50_ values were interpolated from curves generated in GraphPad Prism (v8.4.2) using the four-parameter ‘[Inhibitor] vs. response’ analysis module with the bottom constrained to > 0 and top to = 1.

### Invasion assay

This assay was developed from that published by^94^. Briefly, HSJD-DIPG-007 cells were seeded as bulk spheroids. The following day the growth medium was supplemented with a first dose of the GI_40_ or GI_70_ of LLY-283 or a DMSO vehicle control. Two days later cells were split and reseeded as individual spheroids in an ultra-low-attachment 384 well plate, with the same final media concentration of LLY-283 or vehicle control (i.e. a second dose). The following day half of the spheres were embedded in a final concentration of 4.65 mg/mL Matrigel (again supplemented with a third dose of LLY-283 or DMSO control) and half were maintained without Matrigel to control for the increase in size caused by proliferation.

Spheres were imaged in brightfield with a Nexcelom Celigo before addition of Matrigel® and at 72 h. The overhead cross-sectional area of each sphere was scored by Nexcelom analysis software. Invasion was quantified as (cross-sectional area of Matrigel embedded spheres)/(cross-sectional area of unembedded controls).

### Limiting dilution assay

5,000 (vehicle) or 27,500 (+ LLY-283) HSJD-DIPG-007 cells were seeded as loose spheres. The following day a single dose of the vehicle control or the LLY-283 GI_70_ was added in a 1:10 dilution. After a 7-day treatment period cells were dissociated, passed through a 0.2 µm strainer and live cells were counted in (i.e. trypan blue staining cells were discounted). Cells were re-seeded at a density of 9 cells/well in 6 technical replicates without any LLY-283 or vehicle, then serially diluted by 1.5 × across the plate, to densities of 6, 4, 2.7, 1.8, 1.2, 0.79, 0.53, 0.35 and 0.23 cells/well, each with 6 technical repeats. After 7 days, clones were visualised with Calcein AM (2 µM in PBS, incubated for 30 min at 37 °C) (Sigma-Aldrich, C1359) and imaged with the Nexcelom Celigo image cytometer. Clones were called using Nexcelom analysis software with the ‘Colony 1’ algorithm, and the minimum colony size limited to 2000 µm^2^. The fraction of wells which had clones at each concentration for each cell line in each independent repeat was input to the ‘elda’ function (R, statmod package, v1.4.36) and a Poisson distribution describing the proportion of clonogenic cells (with 95% CI) in each independent repeat was estimated. Mean clonogenic cell fractions were compared between groups by unpaired t-test.

### Immunoblotting

HSJD-DIPG-007 cells were seeded, and the following day treated with a DMSO vehicle control, LLY-283 GI_40_ or GI_70_. After the indicated growth period, cell protein was harvested in RIPA buffer, with complete protease inhibitor cocktail (Roche, 11,697,498,001). Lysate was treated with 0.5 U/µL benzonase (Sigma, E1014) for 20 min at 4 °C to remove DNA, clarified by centrifugation, and protein concentration normalised by DC protein assay (5,000,111, Bio-Rad).

Immunoblotting was performed according to standard protocols. Primary antibodies were anti-Sm B/B’/N (Santa Cruz, sc-130670, 1:200), anti-SDMA (Cell Signalling Technologies, #13,222, 1:1000) and anti-GAPDH (Cell Signalling Technologies, #97,160, 1:2000). Secondary antibodies were anti-rabbit and anti-mouse (Dako, P0260 or P0448, 1:5000). Blots were stained, stripped using an acidic stripping buffer (200 mM glycine, 35 mM SDS, 1% Tween-20 in diH_2_O) for 1 h at room temperature and re-stained in the following order: anti-S_m_ B/B/N, SDMA, GAPDH. Blots were imaged digitally with an Alliance 4.7 (UVITEC) and quantified in ImageStudio Lite® (v5.2).

### Cell death assay

175 SU-DIPG-IV cells were seeded per well in 4 technical replicates in flat-bottomed 384 well plates. The following day serial dilutions of LLY-283, GSK591, M4K2009 or M4K2163 and 10 µg/mL PI (final concentration 1 µg/mL) were added as a single dose in 0.1 volumes of culture medium. After 7 days of growth with compounds, fluorescence intensity due to PI staining of dead cells was recorded with a ClarioSTAR plate reader (2 mm spiral scan). PI intensity was normalised to the vehicle control for each compound before graphing in GraphPad Prism. Points are the mean of three independent repeats, bars are SD.

### RNA-sequencing

HSJD-DIPG-007 cells were cultured for 0, 1, 2, 3, 5 or 7 days + /− GI_40_ LLY-283. Growth medium (supplemented with a second dose of the same concentration of LLY-283 or vehicle control) was replaced after 5 days. Total cellular RNA was harvested with the RNeasy Mini Kit (Qiagen, 74,104), including on-column DNase digestion with RNase-free DNase (Qiagen, 79,256). RNA quality was assessed with an Agilent Tapestation 4150 (all samples had a RNA integrity number of > 9.9). cDNA libraries were synthesised from 200 ng RNA using an NEBNext Poly(A) mRNA Magnetic Isolation Module and NEBNext® Ultra™ II Directional RNA Library Prep Kit (NEB #E7760) according to manufacturer’s instructions. Libraries were quantified with an Agilent D1000 high sensitivity screen tape on an Agilent Tapestation 4150 and pooled at equimolar concentrations. Paired-end sequencing (41 bp reads) was performed with a NextSeq500 platform (Illumina). Raw sequence information was stored as FASTQ files, and trimmed reads were pseudoaligned to the human transcriptome (hg38) with kallisto^95^.

Differential transcript expression was assessed using DESeq2 with a BH adjusted *p* (p.adj) value < 0.05^96^. A Wald test was used for gene ontology analysis and volcano plots, whereas likelihood ratio testing was used for time course analysis. Log fold change is expressed as LLY-283 treated (numerator) versus vehicle treated (denominator) samples.

Gene set enrichment analysis was performed on a list of 603 differentially expressed genes with p.adj < 0.05 and absolute log_2_(fold change) > 0.5, with the clusterProfiler package^97^ and the KEGG database^98–100^. Only GO:BP annotations were considered. Lists of enriched GO terms were shortened by combining similar terms using the ‘simplify’ function with a cut-off value of 0.7.

Analysis and plots were generated in RStudio (v2021.09.0).

### qPCR

HSJD-DIPG-007 cells were treated with a single dose of the LLY-283 GI_70_, GI_40_, or a 0.02% DMSO vehicle control for 0, 3, 5 or 7 days. Total cellular RNA was harvested with the RNeasy Mini Kit (Qiagen, 74,104), and quantified with a NanoPhotometer NP80 (Implen). A cDNA library was generated from 250 ng RNA by reverse transcription with the SuperScript™ IV First-Strand Synthesis System (Invitrogen, 18,091,050). Primers (200 nM each of forward and reverse primers listed in Supplementary Table 5), cDNA (2 ng per reaction) and KAPA SYBR® FAST qPCR Master Mix (KAPA Biosystems, KK4617) were combined and qPCR performed using the Viia7 Real-Time PCR System (Roche) with the following cycle parameters: Enzyme activation at 95 °C for 2 min; 40 cycles of denaturation at 95 °C for 2 s, annealing and extension at 60 °C for 20 s; followed by melt curve generation.

Transcript expression was normalized to the loading control (TATA-Box Binding Protein, TBP) by the delta cycle threshold method (dCt), then to the time = 0 of each independent repeat (as ddCt).

### Animal studies

All in vivo experiments were performed in accordance with institutional and European guidelines (EU Directive 2010/63/EU) and were approved by the local animal care and use committee (Comite Etico de Experimentacion Animal at Universidad de Barcelona, protocol 135/11), and are reported in compliance with ARRIVE guidelines. 4–6 week old female athymic nude mice were anaesthetised with one injection of ketamine (100 mg/kg) and xylazine (10 mg/kg) and 500,000 HSJD-DIPG-007 cells were stereotactically implanted into the fourth ventricle (vehicle treatment n = 15, LLY-283 50 mg/kg n = 15). Animals with neurological symptoms after inoculation of cancer cells, or animals that did not gain weight as expected for normal development were excluded from the study. Mice were randomly assigned to each study arm, whilst ensuring that both treatment arms were represented in each cage, and treated + /− 50 mg/kg LLY-283 daily by oral gavage for 3-days-on-4-days-off, for 4 weeks as previously described^32^. Two hours after the final dose of LLY-283, 5 mice from each treatment group were sacrificed by anesthesia with isoflurane before decapitation. The remaining mice (n = 10 for each group) were used to assess survival with the endpoint criterion being 20% weight loss due to disease. It was estimated that this experiment would detect a minimum increase in survival from 0 to 15% following treatment with a power of 80% (calculated using MedCalc, v20.218, Ostend, Belgium). Median survival for each group was compared by log-rank (Mantel-Cox test, *p* = 0.2).

Tumour burden was assessed in the samples harvested 2 h after the final dose by digital drop PCR (ddPCR) with reporter primers specific to either the mutant or wild-type alleles (Supplementary Table 6). DNA was extracted from FFPE brain tissue with the QIAmp DNA FFPE Tissue kit (Qiagen). ddPCR reactions were added to a DG8 Cartridge for a QX100TM/QX200 Droplet Generator (BIO-RAD). Droplet amplification was performed in a Veriti 96 Well Thermal Cycle (Applied Biosystems), read with a QX200 Droplet reader™ (BIO-RAD), and data were analysed using the Quantasoft Analysis Pro programme. The mass of DNA was calculated as (number of mutant allele positive droplets + number of WT allele positive droplets) * 0.003 ng.

#### Immunohistochemistry

Upon sacrifice, mouse brains were formalin fixed and paraffin embedded. Tissue sections were processed for immunohistochemical staining with the BOND-MAX autostainer (Leica, Germany). Antigen retrieval was performed at 100 °C for 20 min using Epitope Retrieval Solution 1 (AR9961, Leica). Primary antibody or IgG control incubation was for 1 h and signal detected using the BOND™ Polymer Refine Detection System (DS9800, Leica) according to manufacturer’s instructions. Primary antibodies were anti-SDMA (Cell Signalling Technologies, #13,222, 1:200), anti-H4R3me2s (EpiGentek, a-3718, 1:150), anti-human nuclear antigen (Sigma-Aldrich, MAB4383, 1:200). Isotype controls were mouse IgG (1:150, ReliaTech IgG-010) or mouse IgG1 (1:500, Cell Signalling Technology #5415). Images were analysed using FIJI/ImageJ (v1.53c), and the Trainable Weka Segmentation plugin (v3.2.34)^101^ was used for segmentation of the images.

### Supplementary Information

Supplementary Information.

## Data Availability

The RNA-sequencing datasets reported in this manuscript have been deposited the Gene Expression Omnibus under Accession Number: GSE230065.
