# Supplementary material for: PRMT5 inhibition shows in vitro efficacy against H3K27M-altered diffuse midline glioma, but does not extend survival in vivo
Source: Sci Rep. 2024 Jan 3;14:328. doi: 10.1038/s41598-023-48652-x (PMC10764357; doi:10.1038/s41598-023-48652-x)
Supplement: Supplementary file 1 — Supplementary Information. [file 41598_2023_48652_MOESM1_ESM.pdf]

PRMT5 inhibition shows *in vitro* efficacy against H3K27M-altered diffuse midline glioma, but does not extend survival *in vivo.*

Elizabeth J Brown[1], Leire Balaguer-Lluna[2], Adam P Cribbs[3,4], Martin Philpott[3,4], Leticia Campo[5], Molly Browne[5], Jong Fu Wong[1], Udo Oppermann[3,4], Ángel M Carcaboso[2], Alex N Bullock[1†*], Gillian Farnie[1,4†*‡]

[1]Centre for Medicines Discovery, Nuffield Department of Medicine, University of Oxford, Oxford, UK

[2]SJD Pediatric Cancer Center Barcelona, Hospital Sant Joan de Deu, Institut de Recerca Sant Joan de Deu, Barcelona, Spain

[3]Botnar Research Centre, Nuffield Department of Orthopedics, Rheumatology and Musculoskeletal Sciences, National Institute of Health Research Oxford Biomedical Research Unit (BRU), University of Oxford, Oxford, UK

[4]Oxford Centre for Translational Myeloma Research, University of Oxford, Oxford, UK

[5]Experimental Cancer Medicine Centre, Department of Oncology, University of Oxford, Oxford, UK

[†]These authors share last authorship

[‡]Present address: Cancer Research Horizons, The Francis Crick Institute, London, UK

[*]Corresponding authors: alex.bullock@cmd.ox.ac.uk; gillian.farnie@cancer.org.uk

Supplementary Material

Supplementary Table 1 – Full list of SGC epigenetic probes used in the DMG viability screen

Additional information on the SGC probe set can be viewed on the SGC website (https://www.thesgc.org/chemical-probes/epigenetics)

Probe name	Domain[1]	Protein Target	*in vitro* IC(50)or Kd (nM)	PubMed ID	Negative control
(R)-PFI 2 hydrochloride	MT	SETD7	2.0 ± 0.2	25136132	(S)-PFI 2 hydrochloride
A 196	MT	SUV420H1/H2	21 ± 3		SGC2043
A 366	MT	G9a/GLP	3.3/38	24900801	
A 395	WDR	EED	34		A-395N
A 485	HAT	p300/CBP	10/3	28953875	A 486
BAY 299	BRD	BRPF2/TAF1	BRPF2: 67; TAF1 (2nd): 14		BAY-364
BAY 598	MT	SMYD2	27	27075367	BAY-369
BAY 6035*	MT	SMYD3	88±16		
BAZ2-ICR	BRD	BAZ2A/2B	130/180	25719566	
BI 9564	BRD	BRD9/7	14/239	26914985	
GSK 2801	BRD	BAZ2A/2B	257/136	25799074	GSK8573
GSK 343	MT	EZH2	4	24900432	
GSK 591 dihydrochloride	MT	PRMT5	11	26985292	SGC2096
GSK 6853	BRD	BRPF1B	8	27326325	GSK9311
GSK 864*	DEHYD	IDH1 R132C/R132H/R132G	R132C/R132H/R132G: 9/15/17	26436839	
GSK 8814	BRD	ATAD2A/B	50	27530368	GSK 8815
GSK J4	KDM	JMJD3/UTX	GSK-J1: 60	22842901	GSK J5
GSK LSD 1 dihydrochloride	KDM	LSD1	16	26175415	
I-BRD9	BRD	BRD9	50 ± 17	25856009	
I-CBP 112	BRD	CREBBP/EP300	151/625	26552700	
JQ-1	BRD	BET family	BRD4 1st/2nd BRD: 50/90	20871596	(-)-JQ1
L Moses dihydrochloride	BRD	PCAF/GCN5	PCAF/GCN5: 126/600 (ITC)	27966810	D-Moses
LLY-283*	MT	PRMT5	22	26985292	SGC2096
MS 023 dihydrochloride	MT	PRMT type 1	PRMT1/3/4/6/8: 39/135/93/4/5	26598975	MS094
MS049 oxalate salt	MT	PRMT4/6	PRMT4/6: 44/63	27584694	MS049N
NI 57	BRD	BRPF1/2/3	31/108/408		
NVS-CECR2-1	BRD	CECR2	8		
OF 1	BRD	BRPF1/2/3	100/500/2400		
OICR 9429	WDR	WDR5	64	26167872	OICR-0547
PFI 3	BRD	SMARCA2/4	SMARCA4:89; PB1 (5th):48	26139243	BDF25488524
SGC 0946	MT	DOT1L	0.3 ± 0.1	23250418	SGC 0649
SGC 707	MT	PRMT3	31 ± 2	25728001	XY-1
SGC-CBP30	BRD	CREBBP/EP300	21/38	24946055	BDOIA513
SGC-iMLLT	YEATS	MLLT1/3			YT26870
TP 064	MT	PRMT4	< 10		TP-064N
TP 472	BRD	BRD9/7	33/340		TP 472N
UNC 0642	MT	G9a/GLP	< 2.5	24102134	
UNC 1215	MBT	L3MBTL3	40	23292653	UNC 1079
UNC 1999	MT	EZH2/H1	10/45 ± 3	23614352	

[1]BRD, bromodomain; DEHYD, dehydrogenase; KDM, lysine demethylase; MT, methyltransferase; WDR, WD40-repeat.

Supplementary Table 2 – Non-SGC small molecule inhibitors included in DIPG viability screen

Probe name	Domain/complex	Target
PRT4165	PRC1	RNF2/RING1A
UNC3866	PRC1	CBX4/7
SAHA	HDAC	Broad spectrum
SPIN1	Kme	SPIN1
EPZ 6438	PRC2	EZH2

Supplementary table 3 – Characteristics of patient-derived DMG cell lines

MIS = missense; AMP = amplification; TRUNC = truncation

Cell line	Diagnosis	Age	Sex	Source	Histone status	ACVR1 status	TP53 status	EGFR status	Other mutations
**HSJD-DIPG-007**	DIPG	10	M	Autopsy	H3F3A K27M	R206H	WT	WT	PIK3CA MIS[1]
**SU-DIPG-XXI***	DIPG	7	M	Autopsy	HIST1H3B K27M	G328W	Unknown*	Unknown*	-
**SU-DIPG-IV**	DIPG	3	F	Autopsy	HIST1H3B K27M	G328V	WT	WT	MDM4 AMP[2]
**HSJD-DIPG-011**	DIPG	3	F	Biopsy	H3F3A K27M	WT	WT	WT	-
**HSJD-GBM-002**	Left frontal GBM	14	M	Biopsy	H3F3A G34R	WT	TRUNC	WT	PDGFR MIS
**SU-DIPG-VI**	DIPG	7	F	Autopsy	H3F3A K27M	WT	MIS	WT	-

*Whole exon sequencing not performed

Supplementary Table 4 – KEGG terms enriched in day 5 differentially expressed genes

ID	Description	Gene Ratio	Background Ratio	pvalue	p.adjust	qvalue	Gene count
**hsa04020**	Calcium signaling pathway	21/274	240/8101	5.88E-05	0.0096	0.009	21
**hsa00900**	Terpenoid backbone biosynthesis	6/274	22/8101	6.69E-05	0.0096	0.009	6
**hsa00100**	Steroid biosynthesis	5/274	20/8101	4.36E-04	0.0334	0.031	5
**hsa04072**	Phospholipase D signaling pathway	14/274	148/8101	4.65E-04	0.0334	0.031	14

Supplementary Table 5 - Sequences of qPCR primers

Purchased from Eurofins as unmodified DNA oligos (HPSF purification)

Target	Forward sequence	Reverse sequence
**TBP**	GAGCTGTGATGTGAAGTTTCC	TCTGGGTTTGATCATTCTGTAG
**ACAT2**	CCCAGCCAATGCTTCAGGAAT	AAGCCCACGTTTATCAGCTTC
**HMGSC1**	CTCTTGGGATGGACGGTATGC	GCTCCAACTCCACCTGTAGG
**ABCG1**	ATTCAGGGACCTTTCCTATTCGG	CTCACCACTATTGAACTTCCCG
**DLL1**	GACGAACACTACTACGGAGAGG	AGCCAGGGTTGCACACTTT
**NLGN3**	ACAGTGGTGCTAAACCCGTC	ATTGCCATAACTGGCGAGGAT
**TNFRSF1B**	CACATGCCGGCTCAGAGAAT	CTCACAGGAGTCACACACGG
**MDK**	AATGCTCAGTGCCAGGAGAC	GGCTTGGCGTCTAGTCCTTT
**MATN2**	GTGTCAACACCCATGACTATGC	CATCAGGACCAATGTCCAAGAA
**APOE**	GGACGTCCTTCCCCAGGA	GGACGTCCTTCCCCAGGA
**NGFR**	CCTACGGCTACTACCAGGATG	CACACGGTGTTCTGCTTGT
**EPHB2**	GTGTGCAACGTGTTTGAGTCA	ACGCACCGAAAACTTCATCTC

Supplementary Table 6 – Sequences of ddPCR primers

Name	Sequence
Forward primer	GAATTACCGACACACTCCAACAGT
Reverse primer	CTCTGGTCTTCCTTTTCTGGTACAA
WT reporter (VIC)	AGTGGCTCGCCAGATT
Mutant reporter (FAM)	CAGTGGCTCACCAGATT

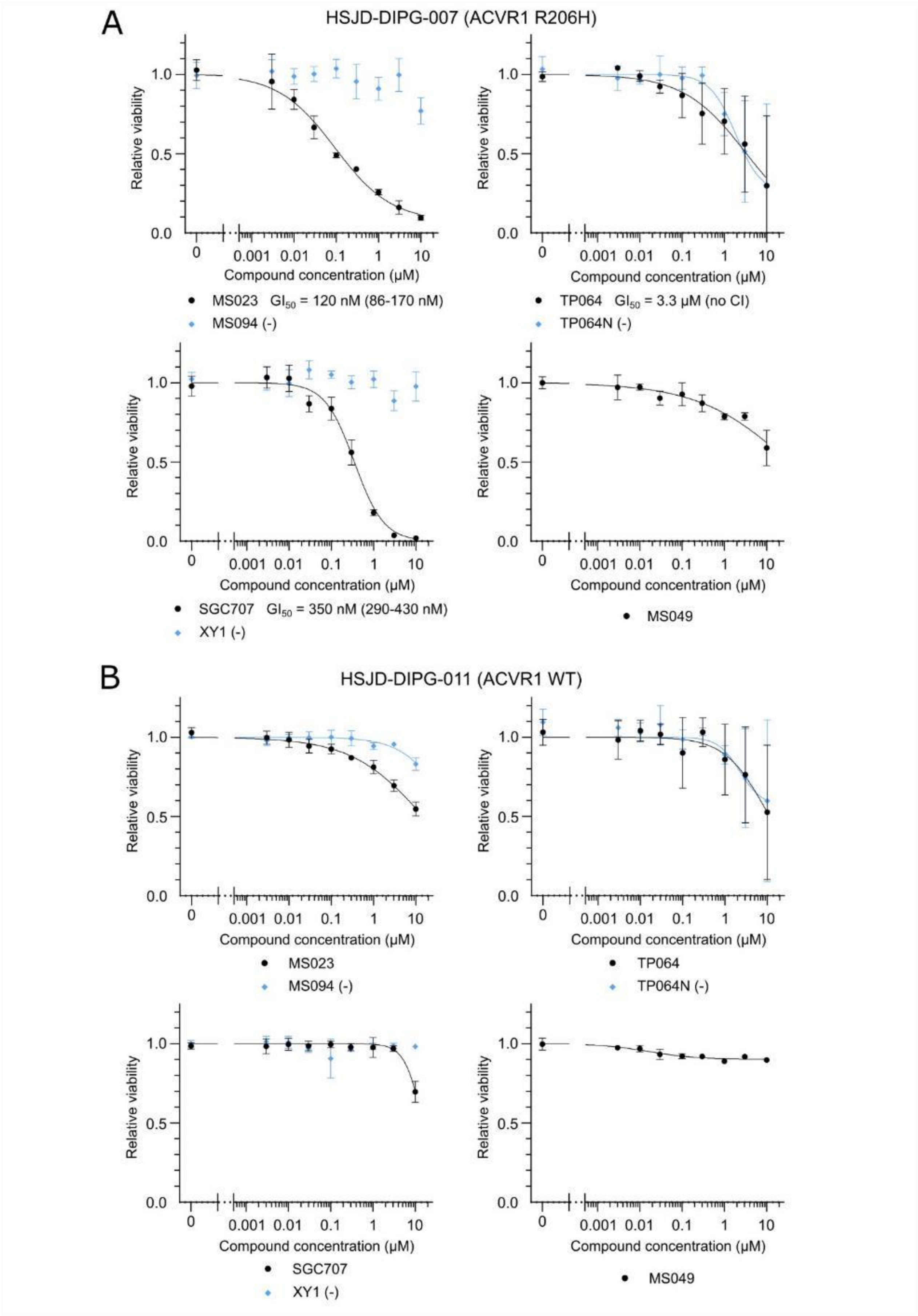
A
HSJD-DIPG-007 (ACVR1 R206H)
Relative viability
Compound concentration (µM)
MS023 GI50 = 120 nM (86-170 nM)
MS094 (-)
TP064 GI50 = 3.3 µM (no CI)
TP064N (-)
SGC707 GI50 = 350 nM (290-430 nM)
XY1 (-)
MS049
B
HSJD-DIPG-011 (ACVR1 WT)
MS023
MS094 (-)
TP064
TP064N (-)
SGC707
XY1 (-)
MS049

Supplementary Figure 1

PRMT5 inhibition most potently reduces the viability of *ACVR1* mutant (HSJD-DIPG-007) and wild-type (HSJD-DIPG-011). The relative viability of HSJD-DIPG-007 **(A)** or HSJD-DIPG-011 **(B)** single spheroids after a 7-day treatment with the indicated PRMT probe (black circles) or its target negative control (blue diamonds). Data points are the mean of 3 independent repeats annotated with the SD. GI_{50} values are included below each graph where they could be calculated by interpolation of the growth inhibition curve.

A

Representative blots shown in Fig. 2B

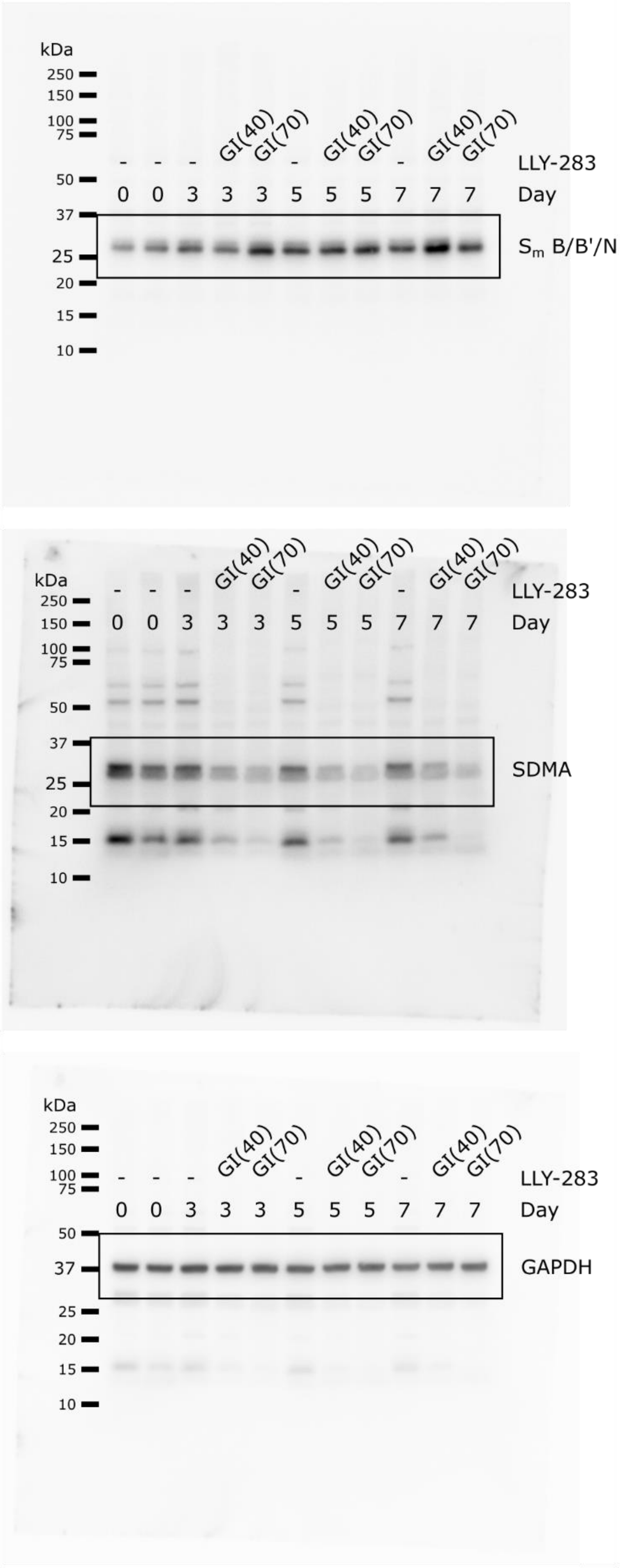

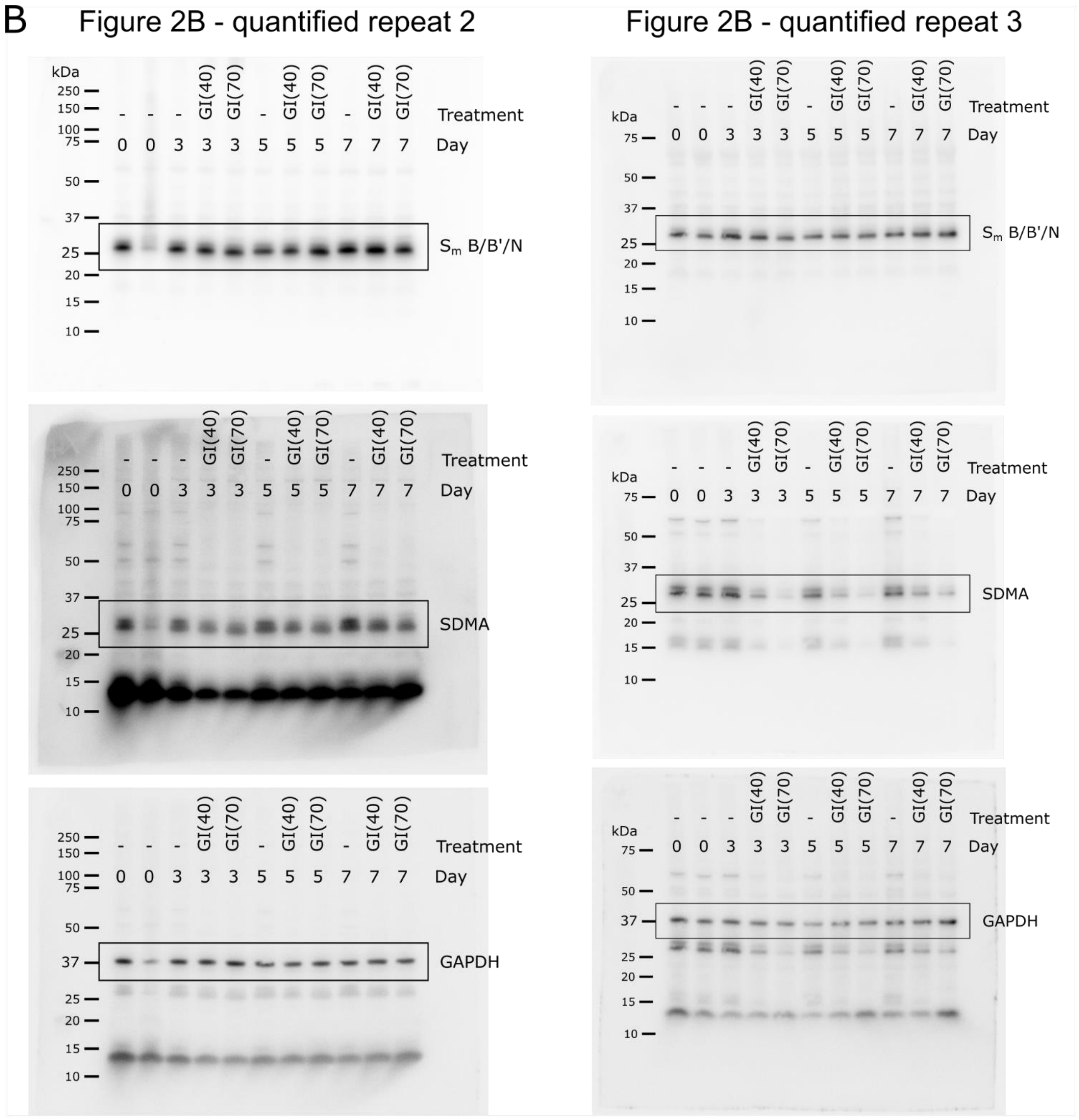

Supplementary Figure 2

(A) Uncropped representative Western blots from Figure 2B (left and right panel). (B) Additional two repeats quantified in Figure 2B (right panel).

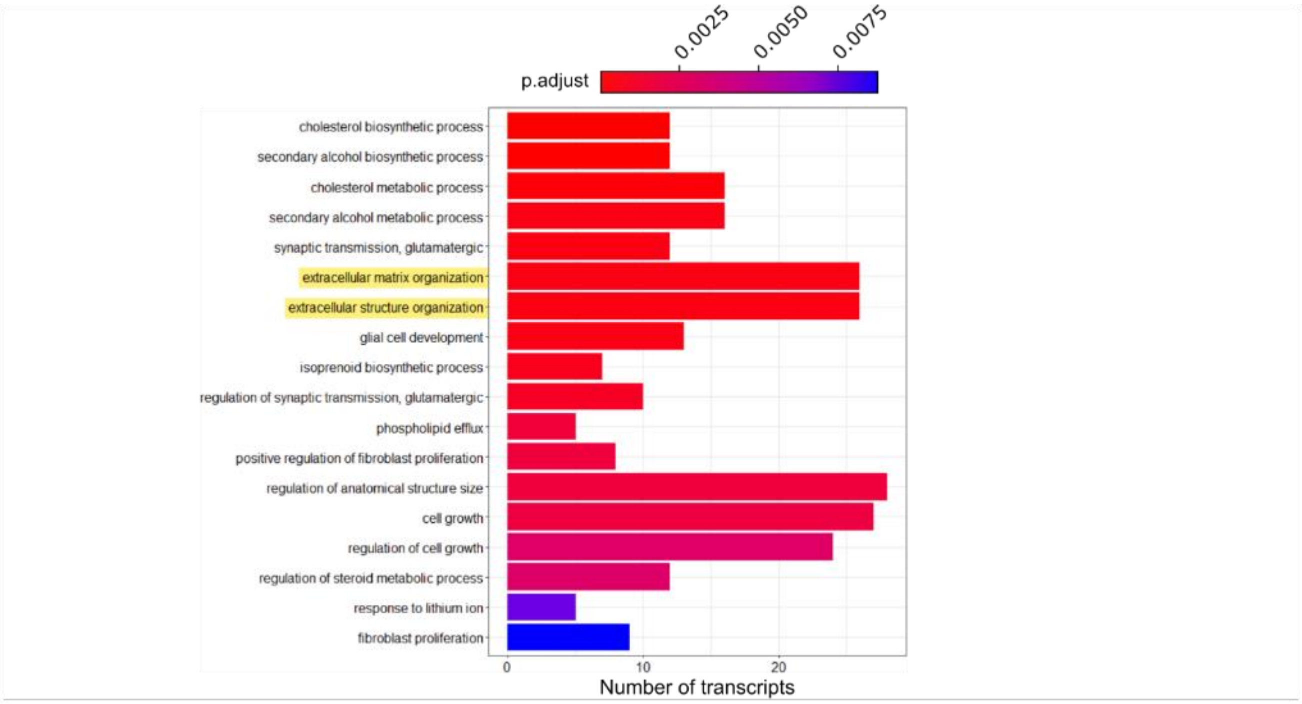

Supplementary Figure 3

The top 25 significantly enriched GO terms in the time course dataset with a ± 0.5 log fold change filter. Redundant GO terms have been omitted. GO terms followed up in subsequent sections are highlighted in yellow. The colour of the bars indicates the p.adj value for the enrichment of that term.

A

Sterol biosynthesis		
Gene	RNA-sequencing	qPCR
APOE	Upregulated	Upregulated
ABCG1	Upregulated	Upregulated
HMGCS1	Downregulated	Downregulated
ACAT2	Downregulated	Downregulated

Gliogenesis/Nervous system development		
Gene	RNA-sequencing	qPCR
MATN2	Upregulated	Upregulated
DLL1	Upregulated	No change
NGFR	Downregulated	Downregulated
MDK	Downregulated	Downregulated
EPHB2	Downregulated	No change
TNFRSF1B	Downregulated	No change

B

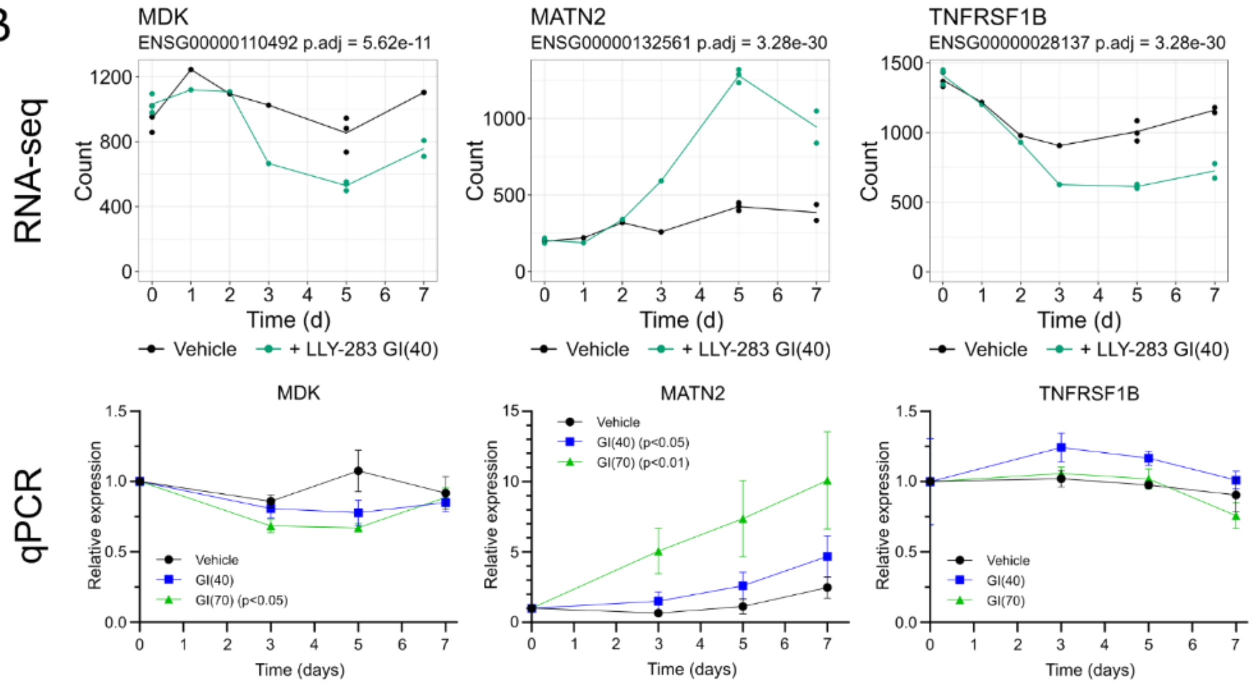

C

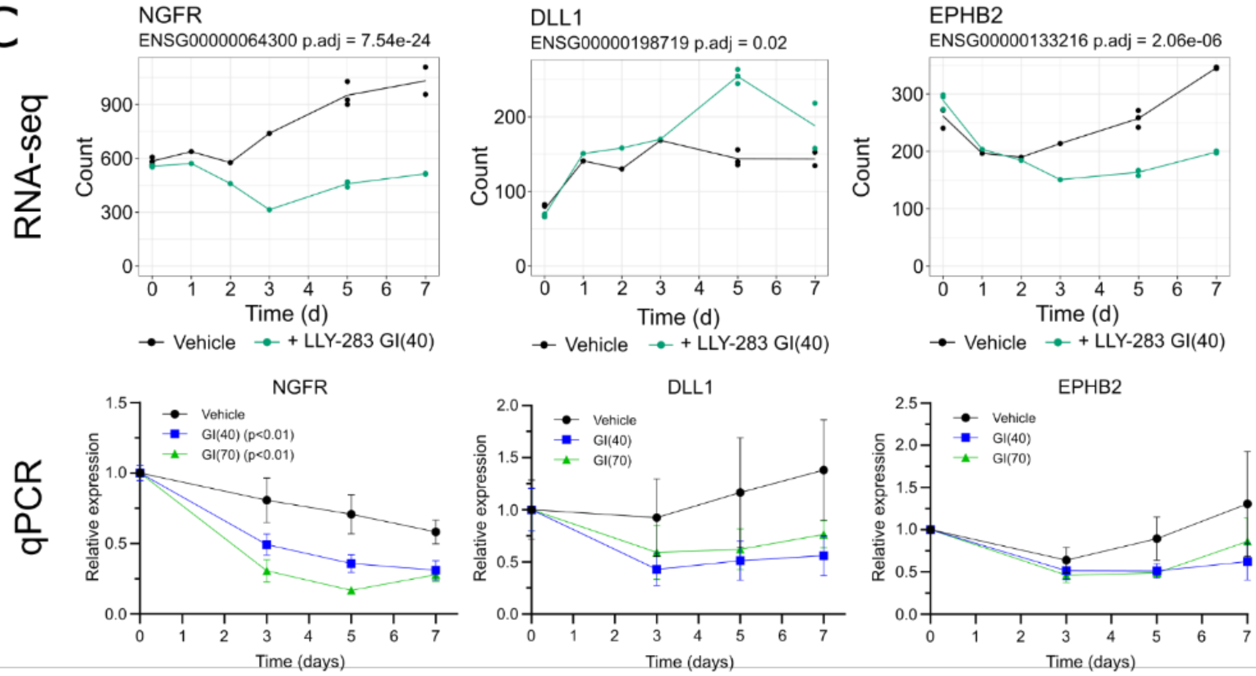

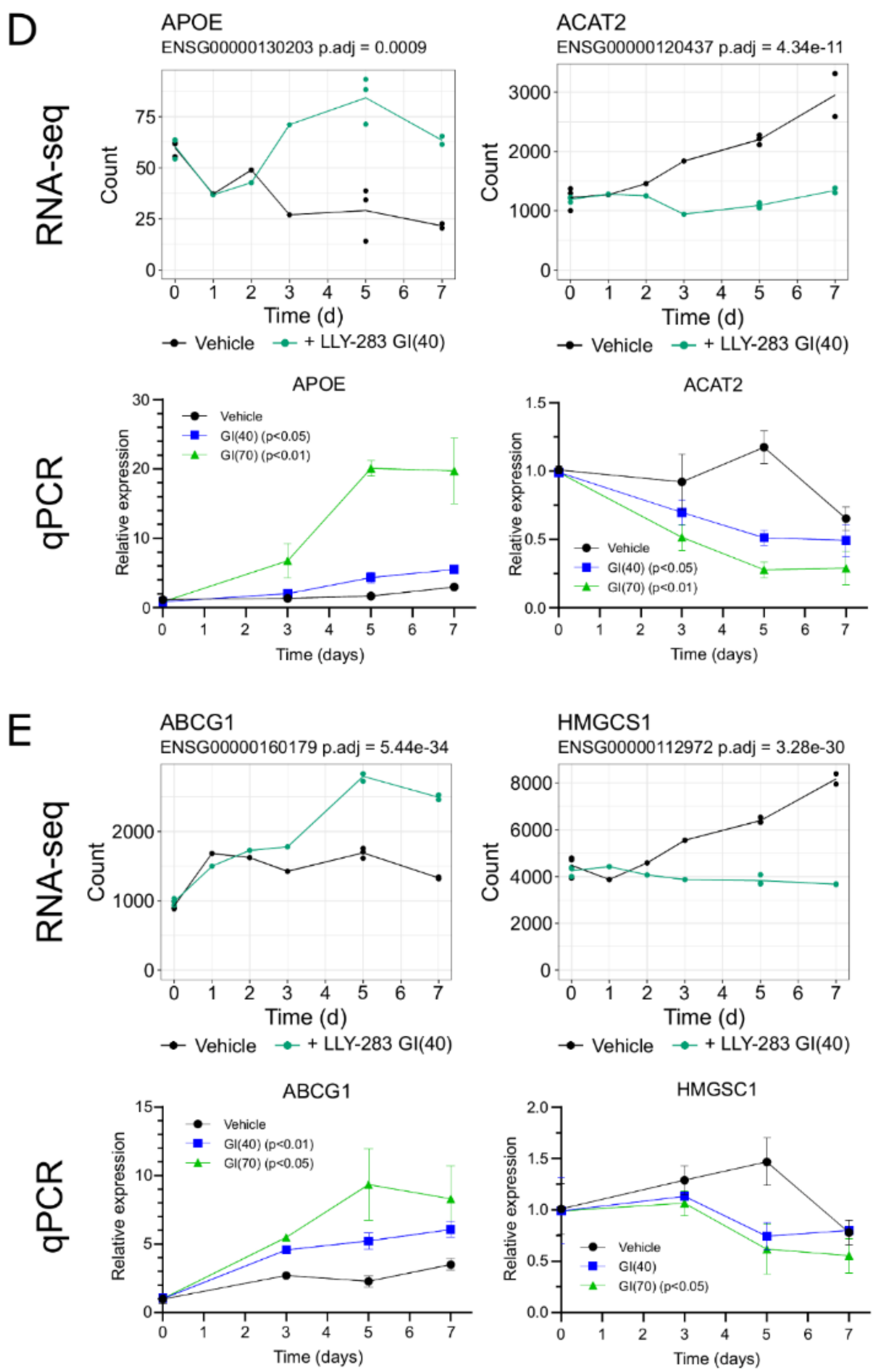
D
RNA-seq
APOE
ENSG00000130203 p.adj = 0.0009
Count
Time (d)
Vehicle
+ LLY-283 GI(40)
ACAT2
ENSG00000120437 p.adj = 4.34e-11
qPCR
APOE
Relative expression
Vehicle
GI(40) (p<0.05)
GI(70) (p<0.01)
Time (days)
ACAT2
E
ABCG1
ENSG00000160179 p.adj = 5.44e-34
HMGCS1
ENSG00000112972 p.adj = 3.28e-30
ABCG1
GI(40) (p<0.01)
GI(70) (p<0.05)
HMGSC1
GI(40)
GI(70) (p<0.05)

Supplementary Figure 4

(A) A summary of the transcripts from each GO term and the differential change observed with RNA-sequencing versus RT-qPCR. The same differential expression after LLY-283 treatment was observed for 7/10 of the transcripts tested. (B-D) (Top rows) Counts of the indicated transcript over time, measured by RNA-sequencing. Adjusted p values were generated by DESeq2. (Bottom rows) HSJD-DIPG-007 cells were treated with LLY-283 GI_{40} or GI_{70} for 0, 3, 5 or 7 days. Transcripts were quantified by RT-qPCR. Points are the mean of three independent repeats. P values were generated by two-way ANOVA, with Dunnett's multiple comparison test between the vehicle and either the LLY-283 GI_{40} or GI_{70} treatments.

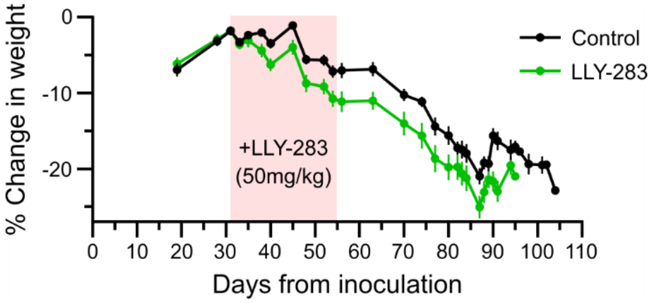

Supplementary Figure 5

PDX mice were treated with either 50 mg/kg LLY-283 or the vehicle control, 3 days on 4 days off, for 4 weeks. Points are the mean weight of all surviving mice, vertical bars are the standard error of the mean. By the final day of treatment, mice treated with LLY-283 experienced moderate weight loss of 11% ± 4% (mean and SD) whereas vehicle controls lost 7% ± 3%. Weight loss was significantly greater in the LLY-283 treated group ($p = 0.0121$; two tailed t-test).

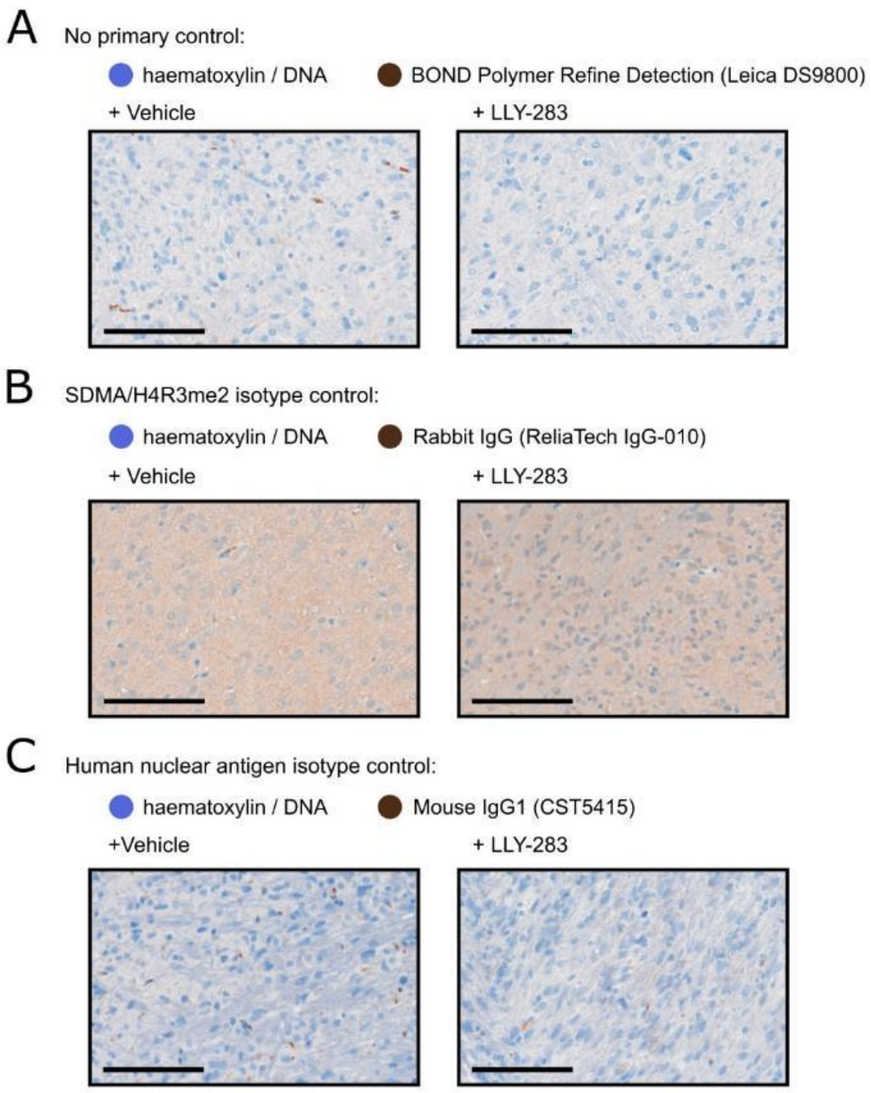

Supplementary Figure 6

Control staining for immunohistochemical experiments. Representative images of mouse brainstems harvested after a 4 week treatment +/- 50 mg/kg LLY-283. Alongside immunohistochemical staining shown in Figure 7 and Figure 8 sections were also treated with the secondary antibody only **(A)** or with polyclonal rabbit IgG at equimolar concentration to the polyclonal anti-SDMA and anti-H4R3me2 antibodies used in Figure 7 **(B)**, or mouse IgG1 at equimolar concentration to the anti-human nuclear antigen antibody used in Figure 8 **(C)**. All are counterstained with haematoxylin. Scale bars represent 100 µm.

1.1 References

1. Mackay, A. *et al.* Integrated Molecular Meta-Analysis of 1,000 Pediatric High-Grade and Diffuse Intrinsic Pontine Glioma. *Cancer Cell* **32**, (2017).

2. Qin, E. Y. *et al.* Neural precursor-derived pleiotrophin mediates subventricular zone invasion by glioma. *Cell* **170**, 845-859.e19 (2017).
